# Quantifying the Rhythm of KaiB-C Interaction for *In Vitro* Cyanobacterial Circadian Clock

**DOI:** 10.1371/journal.pone.0042581

**Published:** 2012-08-10

**Authors:** Lan Ma, Rama Ranganathan

**Affiliations:** 1 Bioengineering Department, University of Texas at Dallas, Richardson, Texas, United States of America; 2 Green Center for Systems Biology and Department of Pharmacology, University of Texas Southwestern Medical Center, Dallas, Texas, United States of America; National Institutes of Health, United States of America

## Abstract

An oscillator consisting of KaiA, KaiB, and KaiC proteins comprises the core of cyanobacterial circadian clock. While one key reaction in this process—KaiC phosphorylation—has been extensively investigated and modeled, other key processes, such as the interactions among Kai proteins, are not understood well. Specifically, different experimental techniques have yielded inconsistent views about Kai A, B, and C interactions. Here, we first propose a mathematical model of cyanobacterial circadian clock that explains the recently observed dynamics of the four phospho-states of KaiC as well as the interactions among the three Kai proteins. Simulations of the model show that the interaction between KaiB and KaiC oscillates with the same period as the phosphorylation of KaiC, but displays a phase delay of ∼8 hr relative to the total phosphorylated KaiC. Secondly, this prediction on KaiB-C interaction are evaluated using a novel FRET (Fluorescence Resonance Energy Transfer)-based assay by tagging fluorescent proteins Cerulean and Venus to KaiC and KaiB, respectively, and reconstituting fluorescent protein-labeled *in vitro* clock. The data show that the KaiB∶KaiC interaction indeed oscillates with ∼24 hr periodicity and ∼8 hr phase delay relative to KaiC phosphorylation, consistent with model prediction. Moreover, it is noteworthy that our model indicates that the interlinked positive and negative feedback loops are the underlying mechanism for oscillation, with the serine phosphorylated-state (the “S-state") of KaiC being a hub for the feedback loops. Because the kinetics of the KaiB-C interaction faithfully follows that of the S-state, the FRET measurement may provide an important real-time probe in quantitative study of the cyanobacterial circadian clock.

## Introduction

Cyanobacteria is the only prokaryote that exhibit circadian rhythm and the cyanobacterium *Synechococcus elongatus* has emerged as a powerful model system in circadian study [1], [2], [3], [4], [5]. Posttranslational interactions among three proteins, namely KaiA, KaiB and KaiC, are responsible for the generation of ∼24 hr periodic oscillation in *Synechococcus elongatus* [6], [7], [8]. More intriguingly, a self-sustained circadian clock was reconstituted *in vitro* by simply mixing three purified Kai proteins at appropriate stoichiometry, together with ATP [9]. The central protein of the circadian clock – KaiC – is a dual auto-kinase and auto-phosphatase [10], [11], [12], with its four phospho-forms evolving in a cyclic fashion during the ∼24 hr circadian cycle [13], [14], [15]. The other two Kai proteins assist with the progression of KaiC phosphorylation cycle: protein KaiA enhances the phosphorylation activity of KaiC while protein KaiB antagonizes such effect [7], [10].

The cyanobacterial circadian machinery is regulated by the phosphorylation cycle of KaiC as well as the interactions among Kai proteins. Using the *in vitro* KaiABC oscillator, several groups have studied diverse aspects of KaiC phosphorylation, including its circadian rhythm [9], [16], robustness under varying protein stoichiometries [17], and dependence of periodicity on mutations [18]. However, the interaction between KaiC and the other two Kai proteins is not fully understood. Experimentally, the existing reports on the interactions among Kai proteins are not consistent. For instance, the complexes formed between KaiC and the other two proteins measured by electron microscopy (EM) have significantly higher amplitude than what was observed using co-immunoprecipitation (co-IP) [13], [14], [16], [17]. Moreover, different oscillation phases of Kai protein interactions relative to phosphorylated KaiC have been reported by experiments using EM and co-IP with different labeling systems [13], [14], [16], [17]. Just recently, an automated real-time method to detect Kai protein interactions using confocal fluorescence correlation spectroscopy (FCS) has been proposed [19], which could overcome the complicated multistep procedures involved and likely the non-trivial errors introduced in the experiments mentioned above. However, one drawback of the FCS approach is the requirement of expensive confocal or two-photon excitation microscopy. More importantly, the average diffusion time of protein complexation needs to be computed using intricate two-component fitting analysis on top of experimental steps [19], making FCS an approach burdened with heavy post-processing.

In terms of mathematical modeling of the Kai clock, several models have been proposed to reproduce the oscillation of KaiC phosphorylation, among which some have included the kinetics of Kai protein interactions [20], [21], [22], [23], [24]. These models with Kai protein interactions all capture the periodic dynamics of KaiB-C complexes, but predict that the KaiB-C complex oscillates either leading or in phase with the oscillation of total phosphorylated KaiC, a result that does not seem consistent with available experimental data [13], [14], [17]. Two relatively complete models that account for detailed reactions of KaiC hexamerization, phosphorylation and interaction have been developed and are adequately consistent with existing experiments [25], [26]. Nevertheless, the inclusion of every KaiC hexamerization step and the complicated conversions between KaiC hexamers and monomers hinder our focus on the understanding of protein-protein interactions.

In this work, we study the quantitative interactions among Kai proteins using a combined theoretical and experimental approach. First, a mathematical model of cyanobacterial circadian clock is proposed, which is expanded upon the Rust *et al* model of orderly phosphorylation mechanism of KaiC [14], and additionally accounts for the association and dissociation kinetics among KaiA, KaiB and the phospho-forms of KaiC. Same as the Rust *et al* model, this model describes monomeric activities while leaving out the KaiC hexamerization steps. Simulations of the dynamics of Kai protein interaction show that the complex formed by KaiB and KaiC oscillates with the same period as KaiC phosphorylation. However, the oscillation of the KaiB-C complex lags that of the phosphorylated KaiC by ∼8 hour. Secondly, to experimentally validate the predictions on the kinetics of the KaiB-C interaction, in particular the phase delay, we develop a novel FRET-based assay. Specifically, Cerulean, a CFP, and Venus, a YFP, are fused to KaiC and KaiB respectively. Oscillation of FRET signal (represented by the ratio of YFP over CFP) is observed for the *in vitro* clock reconstituted using KaiC-Cerulean, KaiA and KaiB-Venus. Quantitative measurements confirm that indeed the oscillation of the FRET signal lags that of the phosphorylated KaiC by ∼8 hr. Because the KaiB-C complexation closely follows the kinetics of the serine phosphorylated-state (S-state) of KaiC, which is a crucial element mediating one positive feedback loop and two negative feedback loops of the oscillator as indicated by our model, the FRET measurement of KaiB-C therefore can serve as a quantification probe to monitor key dynamics of the cyanobacterial circadian oscillator.

## Results

### Modeling *in vitro* circadian rhythm and Kai protein interaction

#### Model rationale

Several mathematical models of *in vitro* Kai clock have been proposed primarily aiming to reproduce the oscillation of the KaiC phosphorylation. They can be classified into two types based on the feedback loops in the signaling network. The first type contains a long negative feedback loop [16], [20], [22], [23]. In these models, KaiC is phosphorylated by KaiA until its phosphorylation level reaches a certain threshold, then KaiB binds to KaiC to inhibit the phosphorylation, thus completing a long negative feedback loop. Such scheme requires that KaiB associates with highly phosphorylated KaiC only, thus the KaiB-C complex oscillates almost in phase with the KaiC phosphorylaiton. This group of models, however, conflicts with the phase delay of the KaiB-C complex relative to the total phosphorylated KaiC implied in experimental reports [13], [14], [16], [17]. In the second type of models, certain regulatory positive feedback pathways were modeled on top of a central negative feedback loop [14], [21], [27]. Nevertheless, the models in [14], [27] do not include the dynamics of Kai protein interactions, while the model in [21] predicts that the oscillation of KaiB-C complexes leads that of phosphorylated KaiC by ∼4 hr, contradicting the existent experiments [13], [14], [16], [17]. Notably, all the models cited above, except for [14], appear before the kinetics of the multiple phospho-forms of KaiC are discovered, and thus do not account for its underlying reactions. The two recent complete models in [25] and [26] are modified based on [22] and [16] respectively, by adding distinct KaiC phosphor-forms to generate consistent kinetics of protein interactions. In this study however, we seek to develop a simplified monomer model without detailed KaiC hexamerzation reactions to address the problem of Kai protein interactions.

**Figure 1:**
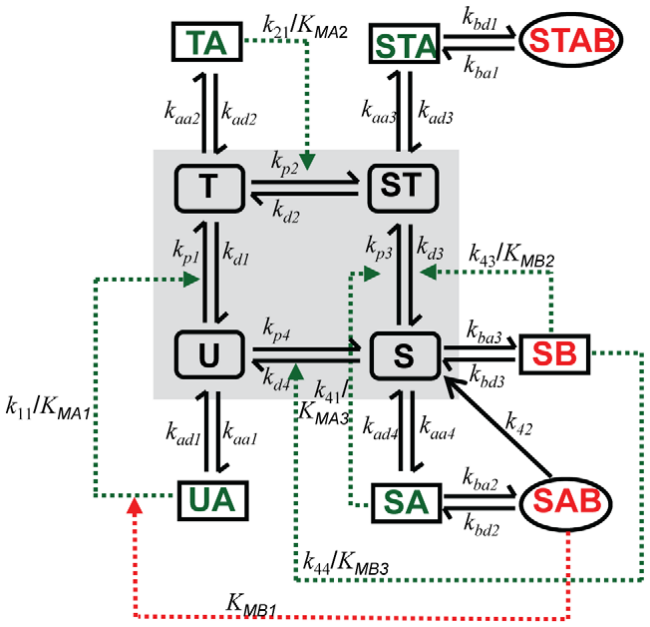
Model scheme of the *in vitro* KaiABC clock. The model includes the ordered phosphorylation/dephosphorylaton among KaiC phospho-forms U, S, ST and S (plotted in shaded box). It also accounts for reactions among the four KaiC phospho-forms, KaiA, KaiB and their derivative complexes. For example, phospho-form T undergoes auto-phosphorylation, auto-dephosphorylation, association and dissociation with KaiA. In addition, UA, TA and SA are assumed to respectively enhance phosphorylation from U, T and S, SB is assumed to promote dephosphorylation from ST to S as well as from S to U, and SAB is assumed to inhibit the phosphorylation from U to T.

**Figure 2:**
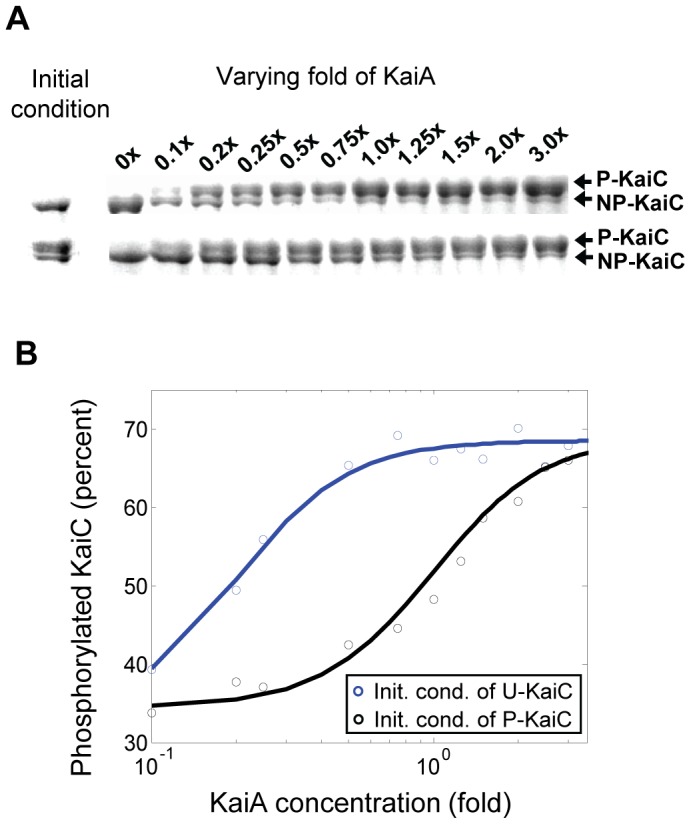
Phosphorylation responses of unphosphorylated or mostly phosphorylated KaiC to varying concentrations of KaiA. (A) KaiC (3.5 µM) of unphosphorylated (upper panel) or mostly phosphorylated (lower panel) initial condition is incubated with different concentrations of KaiA (1× fold equals to 1.5 µM) at 30°C for 16 hrs. Samples at initial condition (left) and 16 hr (right) are subjected to SDS-PAGE using 7.5% gel. (B) Semi-logarithmic plot of relative amount of phosphorylated KaiC at 16 hr as a function of KaiA concentration obtained by quantifying the densitometry of (A) using ImageJ. The data for unphosphorylated (blue circle) and phosphorylated (black circle) initial conditions of KaiC are fitted by sigmoid curves (blue and black lines). Note that the half-maximum concentration of KaiA for unphosphorylated initial condition is about 5 times that for highly phosphorylated initial condition, indicating that KaiA interacts with unphosphorylated KaiC with higher preference.

The Rust *et al* model in [14] provides a proper modeling framework of the orderly conversions among the four phosphor-forms of KaiC monomer, which describes a minimal circadian oscillator of KaiC phosphorylation without explicit protein-protein interaction mechanism. Expanding upon this framework, we propose a model scheme that accounts for not only the recently discovered four phospho-forms of KaiC but also the assembly and disassembly processes among KaiA, KaiB and KaiC (Figure 1). To explain the auto-phosphorylation/auto-dephosphorylation of KaiC and the phosphorylation/dephosphorylation of KaiC regulated by the complexation among KaiA, KaiB and KaiC, the following assumptions of biochemical reactions are proposed based on experiments: first, the phosphorylation process of KaiC conforms to the ordered transition among the four phospho-forms of KaiC [13], [14]. That is, KaiC molecule exists in the states of U, T, S and ST, representing unphosphorylated KaiC, threonine phosphorylated KaiC, serine phosphorylated KaiC and doubly phosphorylated KaiC, respectively. And the cyclic process of phosphorylation and dephosphorylation follows the sequential order of U→T→ST→S→U. Secondly, the binding of KaiA to the four phospho-forms of KaiC are differential such that the probability of the binding for U is the highest, that for T and S are medium, and that for ST is the lowest. This assumption is based on our experiment that evaluates the regulation of KaiC with different phosphorylation level by KaiA (Figure 2). Our data shows differential steady-state phosphorylation level of KaiC depending on its low or high initial phosphorylation condition in response to intermediate concentration of KaiA. In particular, KaiC with unphosphorylated initial condition gets highly phosphorylated by less amount of KaiA than KaiC with mostly phosphorylated initial condition. It is noteworthy that partially phosphorylated KaiC can reach higher phosophorylated state under stimulation by KaiA has been observed before [10]. The data indicates that KaiA preferentially interacts with KaiC of relatively low phosphorylation level. In addition, we assume that once KaiA binds with the KaiC phospho-forms U, T or S, the binary complex subsequently enhances the phosphorylation rate of the respective phospho-form [10], [12]. Thirdly, to implement the antagonization of the KaiA activity by KaiB in regulation, we assume that KaiB sequesters the effect of KaiA by first competitively binding with the complex of KaiA-KaiC phospho-form and then restoring it to free KaiC phospho-form as KaiA and KaiB fall off. And such sequestering and displacement processes are lumped into one reaction rate due to lack of detailed experimental evidences. Noteworthily, KaiB forms complex with ST and S but not with U and T (Nishiwaki, Satomi et al. 2007), therefore the model only includes the ternary complexes STAB and SAB without UAB and TAB. To emphasize the inhibitory effect by KaiB through the S-state of KaiC based on experiments that the antagonizing effect of KaiB on KaiC phosphorylation is observed prominently after the S-state of KaiC is abundant [13], [14], we assume that the antagonizing conversion from the ternary complex STAB to ST is negligible. In addition, we assume that KaiB binds directly only with the S phospho-form and the resultant binary complex SB promotes the dephosphorylation of ST as well as that of S. Also we assume that the ternary complex SAB exerts additional antagonizing effect on KaiA by inhibiting the activity of UA complex. Note that it is not evident if the SB complex will further bind with KaiA molecule because kinetically the complex formation of KaiA-B-C seems to be slower than that of KaiB-C [28]. Therefore, in the current version of model the conversions between SB and SAB are not included and the probable thermal dynamical imbalance thus incurred, if any, will be offset by shift of other rate constants in the parameter space.

Following the conservation law, sum of the concentrations of the eleven KaiC species equals to the total concentration of KaiC, while sum of the concentrations of free KaiA (or free KaiB) (omitted from Figure 1) and its binary and ternary complexes equals to the total concentration of KaiA (or KaiB). Further details of the model can be found in the Supporting Information.

#### Model simulation results

**Figure 3:**
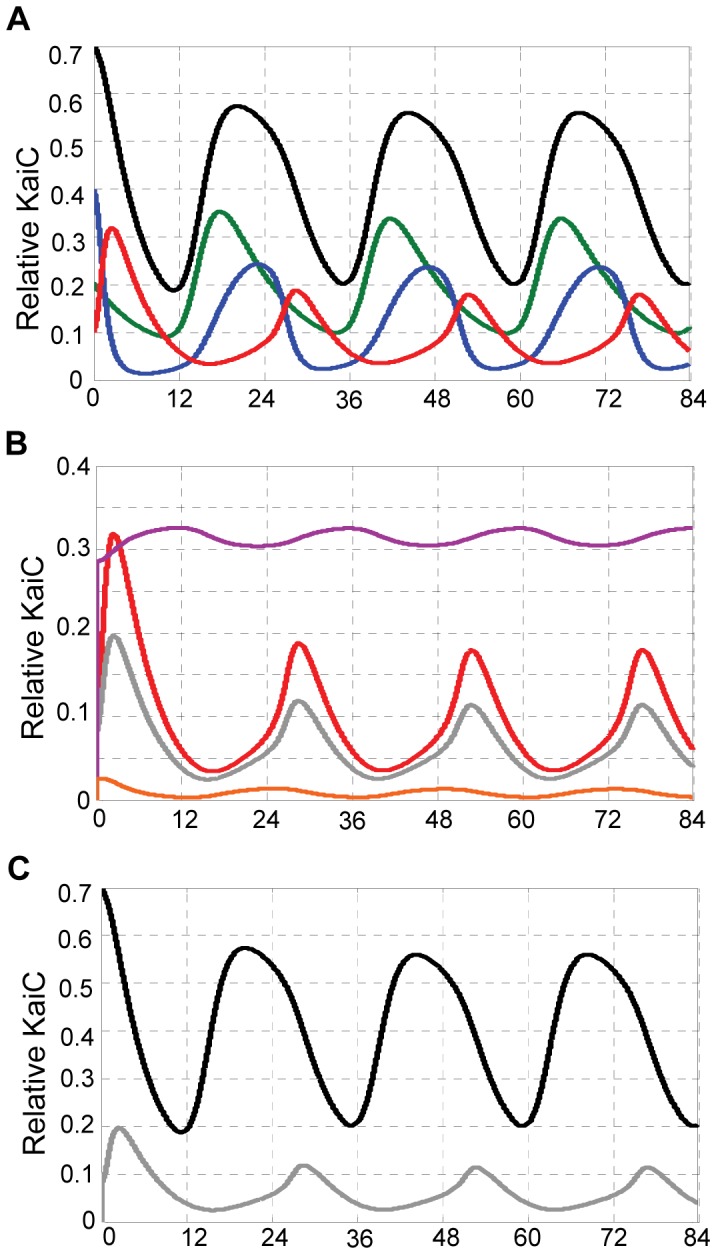
Simulation results of the KaiABC clock model using the initial and total concentrations of KaiA, KaiB and KaiC given in the text. (A) Simulated time courses of T (green line), ST (blue line), S (red line) and total phosphorylated KaiC (black curve) relative to the total amount of KaiC. (B) Simulated time courses of S-phosphorylated KaiC (red line) and complexes of KaiB-C (grey line), KaiA-C (purple line), and KaiA-B-C (orange line), relative to total KaiC. (C) Simulated time courses of phosphorylated KaiC (black line) and complex of KaiB-C (grey line) relative to the total amount of KaiC.

Using the standard concentrations of [KaiC]^T^ = 3.4 µM, [KaiB]^T^ = 3.4 µM, [KaiA]^T^ = 1.3 µM and the initial conditions of KaiC phospho-forms provided in [14], we simulate the model and plot the results of the temporal kinetics of KaiC phosphorylation in Figure 3. Time series of the three phospho-forms of KaiC and the total phosphorylated KaiC replicate the orderly phosphorylation kinetics of T-, ST- and S-states with ∼24 hr periodicity (Figure 3A). Note that the amplitude of the S-state is the smallest and its phase is the latest among the three phosphorylated forms of KaiC, consistent with experimental quantification [13], [14]. Additionally, simulations of Kai protein interactions show that the complex of KaiB-C produces a rhythm with the same period as that of the total phosphorylated KaiC. However, the trajectories of the other two types of KaiC complexes, KaiA-C and KaiA-B-C, are hardly oscillatory (Figure 3B). Since we assume that KaiB molecules bind largely to the S-state of KaiC, the oscillation of KaiB-C complex depends on the amplitude and phase of S-state. As a result, the simulated oscillation of KaiB-C has almost the same phase as that of the S-state (Figure 3B), thus lagging the oscillation of the total phosphorylated KaiC by ∼8 h (Figure 3C). Also, due to the relatively small amplitude of the S-state (less than 20% relative to total KaiC), KaiB-C oscillates with ∼12% amplitude relative to total KaiC (note: the first pulse is not counted due to its transient dynamics) (Figure 3C), which is close to the ∼10% peak-to-trough amplitude relative to the total amount of KaiC measured by Kageyama and co-workers (sum of “BC" and “ABC" in Figure 2F of [17]). Simulation also shows that the amplitude of the complex KaiB-S is much higher than that of the complex KaiB-ST (Figure S1). As a result, the oscillatory dynamics of KaiB-C is mainly contributed by KaiB-S rather than KaiB-ST in terms of phase and amplitude. This agrees with previous experimental findings that KaiB binds to KaiC prominently when the S-state of KaiC is abundant [13], [14]. Overall, the KaiB-C kinetics follows the oscillation of the S-state of KaiC. As we will discuss later, because the S-state of KaiC is a hub for the coupled positive and negative feedback loops imbedded in the model, this important property can be utilized to assay the dynamics of the cyanobacterial circadian oscillator.

#### Model analysis

We further analyze the performance of the model under perturbations of different parameters (see Text S1 for detailed descriptions, and Figure S2 and Figure S3 for stability and bifurcation diagrams). The analysis results show that our model agrees well with the robust performance of the circadian oscillation under variations of the total concentrations of Kai proteins (Figure S2). In particular, when different combinations of two Kai protein concentrations are perturbed, our model adequately captures the robust amplitude and period of oscillation with respect to the changes of KaiB concentration. Our computation also predicts that the variations of the amplitude as well as period with respect to the concentration changes of KaiA and KaiC are linearly correlated; hence the regulation of oscillation amplitude and period could be achieved by tuning either KaiA or KaiC concentration alone albeit with some scaling factor (Figure S2). In addition, the bifurcation diagrams with respect to the variations of basal phosphorylation/dephosphorylation rates indicate that the oscillation of the system exists in wide ranges of parameter values; hence the performance of our model is quite robust (Figure S3). Finally, the stochastic simulations show that the circadian rhythm of the different phospho-forms of KaiC persists quite robustly with respect to the intrinsic noise (see Text S1 and Figure S4).

### Experimental evaluation of KaiB-C interaction

#### Interaction between Cerulean-tagged KaiC and Venus-tagged KaiB produces FRET signal

Because our model indicates that the kinetics of the KaiB-C interaction reflects that of the S-state of KaiC, a potentially critical component for the cyanobacterial circadian network, we propose that an adequate assay to evaluate the coordinated oscillation of the KaiB-C complex will be highly beneficial to the circadian study. It has been discovered that KaiC and KaiB form protein complex *in vitro*, and the amount of the KaiB-C complex measured by a two-step pull-down assay displayed oscillatory kinetics during the *in vitro* clock reaction [17]. Since then, the rhythmic dynamics of KaiB-C interaction have been verified by other methods such as co-IP, EM and BN-PAGE [13], [14], [16], [22]. However, the KaiB-C oscillations measured by these experimental techniques exhibit quite different phase. For instance, the experiments in [13], [14] indicate that KaiB-C oscillate in phase with the S-state of KaiC and there is an 8–9 hr time delay between the oscillations of KaiB-C and the total phosphorylated KaiC, whereas the experiments in [16], [17], [19] show that the phase lag is 4–5 hr. We speculate that the origin of these differences may be caused by the non-trivial errors introduced during the complicated processes involved in these experimental approaches.

In this work, we evaluate the dynamics of KaiB-C interaction by developing a novel FRET assay. We propose to tag Cerulean (an improved cyan fluorescent protein [29]) and Venus (an improved yellow fluorescent protein [30]) to KaiC and KaiB respectively and use the FRET signal produced between KaiC-Cerulean and KaiB-Venus as a measure of the degree of interactions between KaiB and KaiC. Note that KaiC is a relatively big protein with the C-terminal region interacting with KaiA and KaiB [28], [31], [32]. KaiB is the smallest of the three Kai proteins and the existing data indicates that the C terminus of KaiB impedes the interaction between KaiA and KaiC [33]. Based on the above knowledge, we fuse Cerulean and Venus respectively to the C terminus of KaiC and KaiB. The rationale is when the C terminal regions of the KaiC molecule and the KaiB molecule associate with each other, Cerulean and Venus could be brought as close as possible, thus giving rise to high probability of FRET.

**Figure 4:**
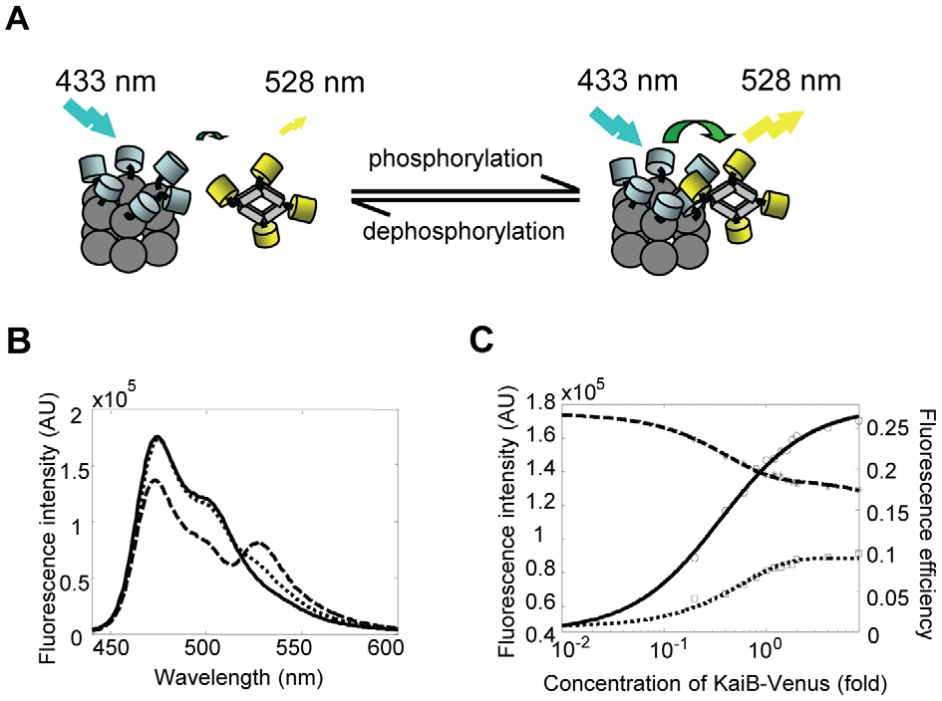
Steady-state FRET between KaiC-Cerulean and KaiB-Venus. (A) Cartoon of interaction between a KaiC-Cerulean hexamer and a KaiB-Venus tetramer. Unphosphorylated KaiC binds with KaiB-Venus at low affinity producing weak FRET, while the S-phosphorylated KaiC recruits KaiB-Venus to its vicinity and induces high FRET signal. (B) Fluorescence spectra of KaiC-Cerulean alone (solid line), KaiC-Cerulean plus KaiB-Venus (dashed line), KaiC-Cerulean plus Venus (dotted line) excited at 433 nm in standard clock reaction buffer. The concentration of KaiC-Cerulean is 3.75 µM. The concentration of KaiB-Venus and Venus is 4.05 µM. Note that the emission spectrum of KaiB-Venus plus Cerulean is almost identical to that of KaiC-Cerulean plus Venus, and therefore is not shown. (C) Peak fluorescence intensities of Cerulean (black dashed line) and Venus (black dash-dot line) plotted versus the concentrations of KaiB-Venus when KaiC-Cerulean (3.75 µM) is incubated with different amount of KaiB-Venus (1× fold equals to 4.05 µM) in clock reaction buffer. The fluorescence intensity is mean of 10 measurements and the standard deviation is included. The FRET efficiency calculated using the mean intensity shows a graded increase (blue line).

It has been shown that one KaiC hexamer binds with one KaiB tetramer during the *in vitro* clock reaction [17], [28], so a bundle of six Cerulean molecules will be brought close to a bundle of four Venus molecules when a KaiC-Cerulean hexamer associates with a KaiB-Venus tetramer (Figure 4A). Whether such a configuration facilitates or hinders the fluorescence energy transfer is unpredictable. Therefore, we test if any FRET occurs by incubating KaiC-Cerulean alone, with Venus or with KaiB-Venus in standard clock reaction buffer and exciting the samples at the wavelength 433 nm (the excitation peak for Cerulean). Note that we use a cuvette with very small path length (Materials and Methods) and ensure that the sample absorbance is less than 0.1 to avoid inner filter effect. As shown in the emission spectrum (Fig. 4B), the fluorescence intensity at 476 nm (the emission peak for Cerulean) is quenched while the fluorescence intensity at 528 nm (the emission peak for Venus) increases when KaiC-Cerulean is incubated with KaiB-Venus, indicating transfer of fluorescence energy. The FRET efficiency at the specific concentration given in Figure 4B is 0.21. This observation of steady-state FRET demonstrates that KaiC molecules could bind with KaiB molecules directly without mediation through KaiA.

For the detection of dynamical protein-protein interactions, the read-out FRET signal must display a magnitude tunable by the degree of intermolecular association. To test if this is the case, we incubate KaiC-Cerulean with different amounts of KaiB-Venus and measure the fluorescence intensity of Cerulean and Venus at their respective emission peaks. Indeed, as the concentration of KaiB-Venus increases, the fluorescence intensity of Cerulean decreases while that of Venus increases (Figure 4C). This means that as more KaiB molecules bind to KaiC, the FRET efficiency becomes bigger (Figure 4C). Such a graded response of FRET to varying amount of KaiB-C complex at steady state provides basis for the detection of dynamical KaiB-C association.

#### CFP and YFP labeled in vitro KaiABC clock exhibits oscillatory FRET signal

**Figure 5:**
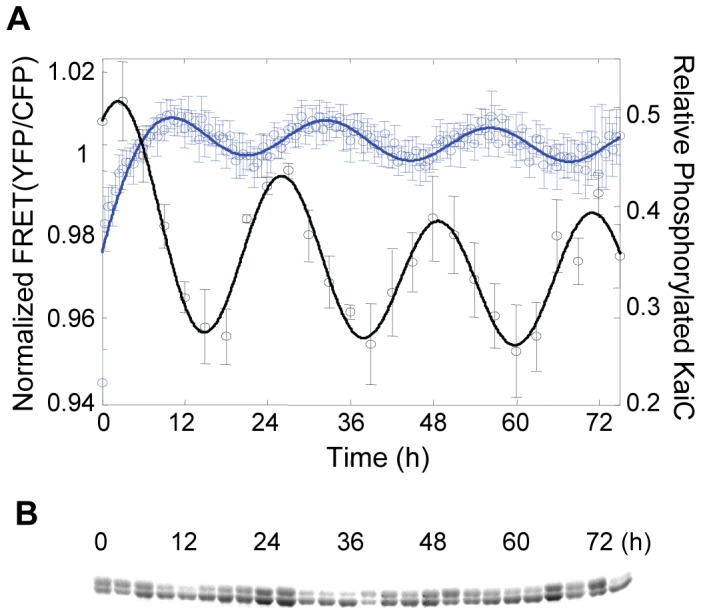
FRET and gel electrophoresis assays of the fluorescent protein-labeled *in vitro* KaiABC clock. (A) Comparison of FRET and electrophoresis assays of the *in vitro* clock. The trajectory of FRET signal (blue circle) is the average of 40 time courses normalized by their respective all-time-point mean. The trajectory of phosphorylated KaiC (black circle) relative to total KaiC is the average quantification of three sets of electrophoresis data including that in (B). The error bars represent the respective standard error. The blue and black lines represent fitted curves of FRET and electrophoresis data. (B) SDS-PAGE gel for the *in vitro* clock of KaiA, KaiB-Venus and KaiC-Cerulean. The protein stoichiometry is described in method. Reaction samples are analyzed on a 6% polyacrylamide gel. The time for each lane at which the sample is taken is labeled on top in hours.

We then reconstitute *in vitro* oscillator using the YFP and CFP labeled KaiB and KaiC proteins together with wild type KaiA. Our titration with Kai proteins shows that the concentrations of KaiA and KaiB-Venus needed for stable circadian rhythm are slightly higher (∼1.1–1.3 fold) than their wild type concentrations, probably because the interactions of fusion proteins are weaker than their wild type counterparts. Based on these stoichiometry guidelines, multiple CFP/YFP-labeled *in vitro* clocks composed of wild type KaiA (1.65 µM), KaiB-Venus (4.05 µM) and KaiC-Cerulean (3.75 µM) are set up in a 96-well plate and excited at 439 nm in a plate reader. The emission intensities of Cerulean and Venus at their respective peaks are recorded every 30 minutes. The fluorescence emission ratio (YFP peak intensity over CFP peak intensity), which is conventionally used in the FRET technology [34], [35], is defined as the final read-out FRET signal. Such ratio also eliminates the significant common noise in both CFP and YFP intensities (Figure S5). Time courses of FRET signals for multiple *in vitro* clocks are measured and normalized by their all-time-point mean values. A final FRET trajectory averaged over 40 individual *in vitro* clocks is plotted in Figure 5A. It reveals a monotonic increase for the first ∼10 hrs, indicating a process of forming KaiB-C complexes once Kai proteins are mixed. After this initial phase, the FRET signal presents stable oscillation with a period of ∼23.5 hr. Note that a similar transient complexation of all three Kai proteins at the beginning of *in vitro* clock has been observed recently [28].

As a control assay, we take time aliquots of the same clock setup as in the FRET experiment and use SDS-PAGE gel electrophoresis to measure the time course of KaiC phosphorylation (Figure 5B). The quantification of phosphorylated KaiC shows that our *in vitro* clock oscillates properly (Figure 5A). In addition, a comparison between the oscillations of FRET and phosphorylated KaiC reveals that the peak of the FRET signal lags that of the phosphorylated KaiC by ∼8 hr (Figure 5A) although two rhythms have the same period. As presented in previous subsection, the static FRET signal positively correlates with the degree of steady-state association between KaiB and KaiC (Figure 4C). In a dynamic situation, where the binding affinity between KaiB and KaiC varies periodically, the temporal change of FRET should then reflect such a rhythm of the KaiB-C interaction. Therefore, the phase difference in Figure 5A indicates that the dynamics of the KaiB-C complex is about 8 hr behind that of the KaiC phosphorylation. This result thus confirms two independent studies using co-IP demonstrating that the KaiB-C complex oscillates in phase with the S-state of KaiC, implying that the KaiB-C interaction has a phase delay of roughly 8–9 hrs relative to the KaiC phosphorylation dynamics [13], [14].

## Discussion

### ATPase activity, monomer shuffling and hexameric interactions of KaiC in the modeling of KaiABC oscillator

It has been shown that the ATPase activity is temperature compensated in WT KaiC and its dynamics is circadian [36], [37]. Although there could exist mutual coupling between the ATPase activity and the kinase activity of KaiC, it is still unclear whether the timing of KaiC phosphorylation is truly dictated by that of the ATPase activity, or rather the ATP hydrolysis is only passively engaged and resonated with the kinase activity of KaiC. Therefore, the ATPase activity of KaiC is considered non-essential for the mechanism of cyanobacterial circadian clock and not included in this model.

The property of monomer exchange among KaiC hexamers has been confirmed by experiments [16], [38]. Computationally it has been integrated into some mathematical models to indicate that the shuffling of KaiC monomer is necessary to maintain synchrony among the KaiC hexamers as well as the high-amplitude oscillations [16], [23], [25]. It is noteworthy that these models that incorporate KaiC monomer shuffling use KaiC hexamers as dynamic variables; that is, the unit molecules that participate in phosphorylation/dephosphorylation and protein interactions are KaiC hexamers. Consequently the exchange of KaiC monomer is required as part of the clock mechanism to synchronize the phosphorylation states of different KaiC hexamers and to achieve stable oscillation. The mechanism of monomer exchange between KaiC hexamers is not explicitly implemented here. Due to lack of evidence showing explicit dependence of the interactions among KaiC and KaiA/KaiB on KaiC hexamer states, our model assumes that the interactions among KaiC monomers and KaiA/KaiB are not differentially dependent on the distinct states of KaiC hexamers and that each KaiC monomer has equal probability to interact with KaiA and KaiB. This assumption implies that the KaiC monomer exchange is decoupled from the phosphorylation process and thus not included in our model of circadian rhythm. Our assumption is indeed supported by previous computational work: Li et al. modeled the KaiABC circadian clock using a multi-layer network of the complete states of KaiC hexamers and showed that the increase in the shuffle rate from 0 µM-1 h-1 to 100 µM-1 h-1 only mildly tuned the periodicity and amplitude of the circadian rhythm, causing the period of KaiC phosphorylation to become longer (from 24 hr to ∼26.5 hr) and the amplitude to decline slightly (∼4% decrease) [24]. In addition, without introducing an explicit monomer-shuffling process, their simulations obtained very consistent results with the experimental study on phase synchronization in [38], indicating that the process of KaiC monomer shuffling may be dispensable for the KaiC phosphorylation mechanism and hence the core model of cyanobacterial circadian clock.

In the circadian experiments, KaiC interact with KaiA and KaiB in hexameric form. Nevertheless, the net effect of hexameric interactions can be abstracted into the rate constants of the monomer model as proposed in [14]. Specifically, using the probabilities of the interactions between KaiA and the KaiC hexamers of different phosphorylation levels as well as the probabilities of distinct KaiC phospho-forms appearing in the KaiC hexamers of different phosphorylation levels, we can obtain qualitative understanding of the probabilities of the interactions between KaiA and the distinct KaiC phospho-forms. For example, it is straightforward to show that, among the binding interactions between KaiA and the four KaiC phospho-forms, the rate for U is relatively high, the rates for T and S are relatively medium, and the rate for ST is relatively low. Such ranking of binding rates has been incorporated into our model. On the other hand, for the interactions between the KaiC phospho-forms and KaiB, our assumptions on probabilities are based on previous experimental observations that U and T do not interact with KaiB, ST has low chance to interact with KaiB, while S has the highest chance to interact with KaiB [13], [14]. Accordingly, our model assumes that U and T do not associate with KaiB, the rate of binding between ST and KaiB is low, and the rate of binding between S and KaiB is high. Furthermore, we would like to point out that the main reason for proposing a monomer model with phospho-form population instead of a hexamer model is because the existing experimental data of KaiC phospho-forms, such as those from gel electrophoresis and co-IP, are mostly given in the format of monomeric activities. In this regard, a monomer model would be more effective in terms of direct experimental evaluation.

### Temperature compensation in KaiABC oscillator modeling

It has been shown experimentally that the *in vitro* KaiABC oscillator is temperature-compensated [9]. In particular, the period of the oscillations decreases by only 10% when the temperature is increased from 25°C to 35°C. Because the phosphorylation and dephosphorylation dynamics of KaiC alone and of KaiC with KaiA show little change between 25°C and 35°C [39], it has been proposed that the period of the oscillator is relatively insensitive to the temperature-induced changes in binding constants, which changes of ∼10 fold from 25°C to 35°C [20]. Following this idea, we test the property of temperature compensation by varying all the dissociation constants by a factor of 3.5 in each direction. Figure S6 shows that the period varies by ∼10%, agreeing with the experimental observations.

### Fluorescent protein tags and interactions of Kai proteins

A potential problem for the clock using KaiC-Cerulean and KaiB-Venus is that the interactions among the three Kai proteins could be affected by the fluorescent tags. So we test if KaiC-Cerulean could be properly phosphorylated and dephosphorylated before running the clock reaction. Gel electrophoresis confirm that after overnight incubation KaiC-Cerulean is dephosphorylated when put in buffer alone or together with KaiB-Venus while KaiC-Cerulean is phosphorylated when put together with wild type KaiA (Figure S7). However, the concentration of wild type KaiA and KaiB-Venus needed for the *in vitro* clock to oscillate are slightly higher than their wild type counterparts, indicating that the fluorescent tags do weaken the interactions, probably by inhibiting the binding affinities between KaiC-Cerulean and the other two Kai proteins. Nonetheless, since the goal of the experiment is to detect circadian oscillations, the validity of the FRET method should be judged by whether the read-out signal oscillates with correct period and relative phase or not, regardless of what the stoichiometry is.

### Dynamics of KaiB-C interaction

It is noteworthy that the binding between KaiB-Venus and KaiC-Cerulean has an initial rising phase of ∼1.5 hrs before settling at steady state (Figure S8), whereas the initial KaiB-C formation process for the *in vitro* clock continues for ∼6–8 hrs (Figure 2B), probably because that the initial association of KaiA to KaiC is quicker and hinders the subsequent binding of KaiB to KaiC. Our model simulations as well as the FRET and electrophoresis data reveal that the trough of KaiB-C complex almost coincides with the peak of phosphorylated KaiC, suggesting that KaiB-C is formed during the dephosphorylation cycle of KaiC. Previous experiments that quantify the KaiB-C complex formation using co-IP demonstrate that the S-state peaks during the KaiC dephosphorylation stage and is almost synchronized with KaiB-C complex [13], [14]. Those data also imply a phase delay of ∼8–9 hrs between KaiB-C complex and the total phosphorylation level of KaiC. We speculate that the S-state of KaiC provides a binding platform for KaiB and its relatively late phase leads to the phase delay between the KaiB-C complex and the phosphorylated KaiC. Our FRET experiments not only show further confirmation on the oscillation and phase of the complex formation between KaiB and KaiC that are described by previous experiments, but also provides a novel means to automatically monitor the S-state of KaiC, a potentially critical component of the cyanobacterial circadian clock as discussed below, in real-time.

### Coupled feedback loops and the role of S-phosphorylated KaiC

The network structure of our model (Figure 1) suggests that the underlying mechanism for the circadian oscillation is the interlinked positive and negative feedback loops. Indeed, perturbation simulations by removing one feedback loop at a time indicate that two groups of feedback loops are indispensible. First, the group of two negative feedback loops of U/T mediated through UA/TA is indispensible, which implements the phosphorylation of U and T promoted by KaiA and is well-recognized as a critical process for the clock mechanism. Secondly, the group of one positive feedback loops and two negative feedback loops is indispensible, all mediated via the S-state of KaiC. When KaiC molecules accumulate in S-state, recruiting more and more KaiB molecules either directly or through KaiA, the resulting KaiB-S complex (SB) dephosphorylates KaiC from ST-state to S-state, forming the positive feedback loop. On the other hand, the SB complex promotes the dephosphorylation of KaiC from the S-state to the U-state, closing a short negative feedback loop. And the SAB complex inhibits the phosphorylation of KaiC from the U-state to the T-state, completing a long negative feedback loop. This group of feedback loops accounts for previous experiments showing that the KaiB-C complex is most abundant only when the S-state of KaiC is abundant and that KaiB promotes the dephosphorylation of KaiC. In summary, aside from confirming a well-accepted observation that the phosphorylation of KaiC promoted by KaiA is important, the indispensible feedback loops indicate that it is critical to assume that the process of KaiC dephosphorylation is mainly regulated by the kinetics of the interaction between KaiB and the S-state of KaiC. In comparison, the minimal model proposed by Rust *et al.* [14] is also based on coupled positive and negative feedback loops [40], but the existence of those feedback loops relies on a relatively strong assumption that KaiA regulates all the reaction rates of KaiC. Although all the regulations by KaiA have been summarized by a biochemically justifiable formula [14], it is not easy to interpret the underlying molecular mechanisms, especially the mechanisms of protein-protein interactions. In our model, all the biochemical reactions such as binding and phosphorylation reactions, are formulated explicitly following the law of mass action or Michaelis-Menten mechanism. The feedback loops thus emerge may better represent the underlying signaling rules.

Because the S-state of KaiC is a hub of the feedback loops, the KaiABC oscillator will be dysfunctional if the S-state is absent. Also, further analysis indicates that the S-state is the core dynamical variable for the model and the performance of the oscillator relies critically on the functioning of the S-state (manuscript in preparation). In this sense, since the kinetics of the S-state is synchronized with that of the KaiB-C complex, our FRET approach can be utilized to evaluate the performance of the *in vitro* clock.

### Limitation of the FRET system

While our FRET sensing system serves as in situ readout of the phase of the rhythm of KaiB-C interaction, it does not provide direct measurement of the amplitude of the rhythm of KaiB-C complexes, as the quantitative relationship between the intensity of FRET signal and the amount of KaiB-C complexes has not been established. Toward this end, one needs to calibrate the observed FRET signal to a direct measurement of complexes formed by KaiB and specific KaiC phospho-forms, possibly using phosphomimetic KaiCs that are mutants designed to act as stable mimics of each of the four KaiC phospho-forms and thus constitutively form stable complexes with KaiB or disrupt KaiB-C interactions [13]. However, as pointed out by a review paper [41], the behavior of different phosphomimetic mutants of KaiC might be different from that of phospho-forms of wild-type KaiC. Indeed, a recent study reveals contradictions between previous experiments conducted using phosphomimetic mutants of KaiC and analysis using wild-type KaiC [25]. And Brettschneider et al. comment that the phosphomimetic mutants might not correctly imitate the true phospho-forms. Due to these concerns of uncertainty in the accuracy of phosphomimetic experiments, we do not perform further calibration experiments of the FRET system in this study and its capacity of reporting the amplitude of KaiB-C oscillation remains to be determined.

### Conclusions

In this study, we expand the minimal model of KaiC phosphorylation proposed by Rust *et al* to a more inclusive model framework that also accounts for the critical dynamics of Kai protein interactions. This model agrees well with existent experimental evidences such as kinetics of KaiC phospho-forms, kinetics of Kai protein complexation, and robustness to protein concentration perturbation. Furthermore, simulations of the KaiB-C interaction show oscillation in phase with the S-state of KaiC while lagging the total phosphorylated KaiC by about 8 hr. To experimentally evaluate the KaiB-C kinetics, we develop a novel FRET-based assay, which confirms the phase difference predicted by our model. As indicated by our model and the existing experimental observations, the serine-phosphorylated KaiC is a key coordinator between the cycling of KaiC phosphorylation and the kinetics of Kai protein interactions. Hence the assay of the S-state of KaiC could help evaluate the functioning of the cyanobacterial circadian clock. As a conclusion, the FRET method developed here is a promising means to quantitatively monitor the *in vitro* KaiABC clock.

## Materials and Methods

### Constructs of fluorescent protein-fused Kai proteins

As a first step of designing the fusion proteins, a nine-residue GGS linker is introduced between the Kai protein and the fluorescent protein to increase flexibility of the fusion protein. Specifically, to generate the KaiC-Cerulean construct, a DNA fragment encoding KaiC is first cloned into pET-28b vector (Novagen) between the NcoI and XhoI digestion sites, where a His6 tag follows the XhoI site. Then a HindIII digestion site is inserted between the C terminus of KaiC and the XhoI site. Finally a DNA fragment encoding GGS linker-Cerulean-Thrombin cutting site is inserted between the HindIII and XhoI sites. The construct of KaiB cloned into pGEX-6P-1 vector is as previously described [42] and is a kind gift from Dr. John Chuang and Dr. Stanislas Leibler of Rockefeller University. To generate the KaiB-Venus construct, an EcoRI site is first inserted between the C-terminus of KaiB and a NotI site right before the stop codon. Then a DNA fragment encoding GGS linker-Venus is inserted between the EcoRI and NotI sites.

### Bacterial expression and purification the Kai proteins

*E. coli* BL-21 (DE3) cells (Novagen) expressing GST-KaiA, GST-KaiB-Venus and KaiC-Cerulean-His6 are grown in TB buffer at 37°C and induced by IPTG at 16°C overnight. The rest of processes are all performed at 4°C. Cell lysis is centrifuged at 20,000×g for an hour. The supernatant of GST-KaiA and GST-KaiB-Venus is incubated with glutathione beads (GE Healthcare) for 40 min. The beads are washed with PBS buffer, equilibrated with PreScission cleavage buffer (50 mM Tris-HCl at pH 7.5, 150 mM NaCl, 1 mM EDTA, 1 mM DTT) and agitated with PreScission protease (GE Healthcare) for 4 hr. Cut KaiA or KaiB-Venus is eluted with PreScission cleavage buffer. KaiA is further purified on a 1 ml Mono Q column (GE Healthcare) by a 0.01–1 M NaCl gradient with 20× column volume. KaiB-Venusias further purified by prep-grade Superdex 200 cloumn (GE Healthcare) in FPLC buffer (20 mM Tris-HCl at pH 8, 0.15 M NaCl, 0.5 mM EDTA, 0.5 mM DTT). Supernatant of KaiC-Cerulean cell lysis is incubated with Ni-NTA beads for 40 min. Then beads are washed by binding buffer (25 mM Tris-HCl at pH 8.0, 0.5 M NaCl, 10 mM Imidazole, 5 mM MgCl_2_, 1 mM ATP) and KaiC-Cerulean-His6 is eluted by elution buffer (50 mM Tris-HCl at pH 8, 0.5 M NaCl, 0.2 M Imidazole, 5 mM MgCl_2_, 1 mM ATP). Eluate is exchanged into HRV3C cleavage buffer (Tris-HCl 50 mM at pH 7.5, NaCl 150 mM, 5 mM MgCl_2_, 1 mM ATP), incubated with HRV3C protease (Novagen) overnight, and incubated with Ni-NTA beads for 40 min. Beads are then washed by HRV3C cleavage buffer and the eluate is collected as cut KaiC-Cerulean. Cut KaiC-Cerulean sample is applied to prep-grade Superdex 200 column (GE Healthcare) in buffer (50 mM Tris-HCl at pH 8, 10 mM NaCl, 5 mM MgCl_2_, 1 mM ATP, 1 mM DTT). All the purified Kai proteins are exchanged into storage buffer (20 mM Tris-HCl at H 8, 150 mM NaCl, 0.5 mM EDTA, 5 mM MgCl_2_, 1 mM ATP, 10% glycerol) and flash frozen at −80°C. Concentrations of the proteins are measured by Bradford assay.

### Reconstitution of *in vitro* circadian clock and electrophoresis assay

The *in vitro* clock is set up by mixing wild type KaiA (1.65 µM), KaiB-Venus (4.05 µM) and KaiC-Cerulean (3.75 µM) in the reaction buffer (20 mM Tris-HCl at pH 8.0, 150 mM NaCl, 0.5 mM EDTA, 5 mM MgCl_2_, 1 mM ATP) at 30°C. To monitor the clock using electrophoresis, a 6.5 µl aliquot of reaction sample is collected every 3 hours, mixed with 6.5 µl blue juice ((2× loading dye from Qbiogene) and stored at −20°C. After 3-day collection, the aliquots are subjected to SDS-PAGE on a 6% gel and the gel is stained by GelCode blue (Pierce Biotechnology). The relative intensity of phosphorylated KaiC is quantified by densitometry using software ImageJ.

### FRET measurement

To obtain a fluorescence emission spectrum, 200 µl reaction sample is loaded into a cuvette with 3 mm light path length (Starna Cells). The cuvette is put into a fluorometer (Photon Technology International) set at 30°C and the sample is excited at 433 nm. The emission spectrum is recorded immediately for KaiC-Cerulean alone or after 30–40 min settling time for KaiC-Cerulean plus KaiB-Venus. Background spectrum of the reaction buffer is subtracted from all plots. Emission spectrum of KaiB-Venus alone is subtracted from the spectrum of mixture of KaiC-Cerulean plus KaiB-Venus and KaiC-Cerulean plus Venus so that only intensity due to FRET is plotted.

To measure the dynamics of FRET signal over time, multiple 100 µl *in vitro* clocks are loaded into a black-wall clear-bottom 96-well plate (Corning Life Sciences). The plate is immediately put in a Wallac 1420 multilabel counter (PerkinElmer) set at 30°C, with excitation filter of 440 nm and two emission filters of 460 nm and 520 nm. Every 30 minutes, the sample is excited and the fluorescence intensities at the two peak emission wavelengths are recorded in a bottom-counting mode for 3 to 4 days.

### Model equations, parameters and robustness analysis

Mathematical equations and parameters of the model are given in Text S1 and Table S1. Stability and robustness analysis of the model are given in Text S1 and Table S2.

## Supporting Information

Text S1 **Modeling the phosphorylation/dephosphorylation and assembly/disassembly of KaiABC oscillator.**
(DOCX)

Figure S1 **Oscillation of KaiB-S and KaiB-ST.** Simulation of the complex KaiB-C (grey), the complex KaiB-S (solid black) and the complex KaiB-ST (dashed black). It shows that the amplitude of the complex KaiB-S is much higher than the amplitude of KaiB-ST. That is, the quantity of KaiB-C is mainly contributed by KaiBC-S rather than KaiBC-ST, which agrees with previous experimental findings that KaiB binds to KaiC only when the S-state of KaiC is abundant.
(TIF)

Figure S2 **The amplitude and period of KaiC oscillation in the parameter planes of Kai protein concentrations.** Simulations of the amplitude and period of the oscillation of KaiC phosphorylation are plotted in different 2-dimensional parameter planes when the total concentrations (unit: µM) of two Kai proteins are varied while the third protein concentration is maintained at the standard condition. Amplitude in (A) [KaiA]^T^-[KaiB]^T^ (B) [KaiB]^T^ -[KaiC]^T^ and (C) [KaiA]^T^-[KaiC]^T^ planes. Period in (D) [KaiA]T-[KaiB]T (E) [KaiB]T -[KaiC]T and (F) [KaiA]T-[KaiC]T planes. In each plot, the heat map represents the amplitude of phosphorylated KaiC relative to the total KaiC concentration, or period (unit: hour) of phosphorylated KaiC, with the corresponding color bar located to the right.
(TIF)

Figure S3 **Bifurcation diagrams of the model of KaiABC oscillator.** In each diagram, steady-state behavior of the system is plotted vs. the parameter under perturbation. Stable oscillation is represented by a pair of circles, indicating the maximum and minimum values of oscillation. Stable equilibrium point is represented by solid line.
(TIF)

Figure S4 **Stochastic simulations of KaiABC oscillator.** The model is simulated by Gillespie algorithm. Shown above are 10 runs of stochastic simulation, where the total phosphorylated KaiC (black), T (green), ST(blue) and S (red) exhibit robust oscillations under noise.
(TIF)

Figure S5 **Time trajectories of donor and acceptor fluorescence intensities during FRET experiment.** Exemplary time trajectories of CFP (blue curve) and YFP (green curve) fluorescence intensities normalized by their respective all-time-point mean values from a FRET experiment. The FRET signal obtained by YFP and CFP ratio normalized by its all-time-point mean value is shown as black solid line. There is significant common noise in the CFP and YFP data, while their ratio eliminates the noise and reveal the signal to a great degree.
(TIF)

Figure S6 **Temperature-compensation of model oscillations.** When the dissociation constants for all binding reactions are simultaneously varied by a factor of ∼10, the period of KaiC phosphorylation changes by ∼10%.
(TIF)

Figure S7 **Phosphorylation test of KaiC-Cerulean with other Kai proteins.** Gel electrophoresis of KaiC-Cerulean incubated alone (lane 1), with KaiB-Venus (lane 2) or with wild type KaiA (lane 3) overnight at 30°C in standard clock reaction buffer. The upper band represents phosphorylated KaiC (P-KaiC) and the lower band represents non-phosporylated KaiC (NP-KaiC).
(TIF)

Figure S8 **FRET trajectory of mixture of KaiC-Cerulean and KaiB-Venus.** Time course of FRET signal when KaiC-Cerulean and KaiB-Venus were incubated in the standard clock reaction buffer at 30°C. The black circles represent an average curve of five normalized FRET trajectories. The black line represents fitted curve of the data. This plot shows that there is an abrupt rise within the first 1.5 hr. After this transient phase, the binding kinetics settles to a stable state.
(TIF)

Table S1 Parameters for the model of KaiABC oscillator.
(DOCX)

Table S2 Oscillation range for the model of KaiABC oscillator. The following model parameters are perturbed one at a time. The lower bound and upper bound of each of the parameters, within which oscillation exists, are listed in the table. The fold range is defined as the upper bound value divided by the lower bound value.
(DOCX)
