# Supplementary material for: Quantifying the Rhythm of KaiB-C Interaction for In Vitro Cyanobacterial Circadian Clock
Source: PLoS One. 2012 Aug 10;7(8):e42581. doi: 10.1371/journal.pone.0042581 (PMC3416856; doi:10.1371/journal.pone.0042581)
Supplement: Text S1 — Modeling the phosphorylation/dephosphorylation and assembly/disassembly of KaiABC oscillator. (DOCX) [file pone.0042581.s001.docx]

**Text S1- Supporting Information**

A model of *in vitro* cyanobacterial KaiABC oscillator that accounts for the underlying phosphorylation/dephosphorylation and assembly/disassembly reactions is described in the main text. We want to emphasize that our model adopts the basic framework of KaiC phosphorylation oscillator from the Rust *et al* model [1], including the inter-conversions among the different KaiC phospho-forms, the inhibition of phosphorylation and sequestration of KaiA by KaiB, and the accelerated dephosphorylation by KaiB, although the Kai protein interactions are expressed as a compact formula in the Rust *et al* model. The model contains 11 KaiC species and the ordinary differential equations governing the dynamics of the 10 independent variables are given below:

where a species name in a pair of square brackets represents the concentration of its monomeric form. The 11th variable S is constrained by the conservation equation: [U] + [T] + [ST] + [UA] + [TA] + [STA] + [SA] + [STAB] + [SAB] + [SB] + [S] = KaiC^T^, where KaiC^T^ represents the total concentration of KaiC molecules. The concentrations of free KaiA and KaiB molecules are represented by [A] and [B], respectively. Their concentrations are constrained by equations: [UA] + [TA] + [STA] + [STAB] + [SA] + [SAB] + [A] = KaiA^T^ and [STAB] + [SAB] + [SB] + [B] = KaiB^T^, where KaiA^T^ and KaiB^T^ represent the total concentration of KaiA and KaiB molecules, respectively.

Next we discuss the biochemical meaning of different terms in the differential equations:

1. The conversions among the four phospho-forms of KaiC, namely U, T, ST, and S, follow the orderly phosphorylation / dephosphorylation principle [1,2]. The basal phosphorylation and dephosphorylation rates are denoted as *k_pi_* and *k_di_* (*i* = 1-4).
2. Free KaiA binds with U, T, ST and S, where the binding probability for U is the highest, that for T and S is medium, and that for ST is the lowest as discussed in the main text. The association and dissociation rates are denoted as *k_aaj_* and *k_adj_* (*j* = 1-4). Experimentally it has been shown that KaiB forms complex with ST and S but not with U and T [2], therefore the model only includes the ternary complexes STAB and SAB without UAB and TAB. In our model, the binary complexes STA, SA as well as S associate with free KaiB, where the association and dissociation rates are denoted as *k_bak_* and *k_bdk_* (*k* = 1-3). Previous experimental data showed that KaiB forms tetramer (or dimer of dimer) and the tetramerization is critical for the maintenance of circadian clock [3,4,5]. To account for this underlying cooperative activity, the binding of KaiB with other molecules is assumed to take a format of fourth order Hill function. Indeed, derivation of the complete model by Brettschneider *et al* indicates that nonlinear dependence on KaiC phosphoforms exists in the reactions and the complexation of KaiC with KaiB has to adopt a power of 5 to achieve desired dynamics (see supporting material of [6]).
3. The phosphorylation of KaiC phospho-form facilitated with KaiA is implemented by a first-order Michaelis-Menten kinetics based on experimental observation [1]. For example, the phosphorylation rate of S promoted by KaiA is assumed to be , meaning that only when abundant KaiA binds to S and the amount of S-KaiA is above the threshold value of that the phosphorylation of S is significantly enhanced.

(4) The inhibition of phosphorylation and the promotion of dephosphorylation by interaction between KaiB and the S-state of KaiC are implemented by an inhibitory Hill function and a regular Hill function, respectively. As stated in the main text, KaiB forms tetramer to interact with KaiC during the *in vitro* clock reaction. To account for the plausible cooperativity induced by KaiB tetramerization [3,5,7,8], we assume a fourth order Hill function. For instance, the phosphorylation rate of U inhibited by the complex SAB is assumed to be , meaning that only when sufficient amount of KaiB binds to the complex of SA and the concentration of SAB increases significantly above the threshold concentration of that the inhibition of phosphorylation exerts its full effect.

(5) It is noteworthy that the direct phosphorylation/dephosphorylation conversions between UA and TA, TA and STA, STA and SA, and SA and UA are omitted in our model. Adding these conversions to the model only introduce slight changes (≤5%) of the period and amplitude of the circadian oscillation (simulations not shown). Therefore we argue that our model without the inter-conversions among the complexes of the KaiA-KaiC phospho-forms is sufficient to explain the phosphorylation dynamics of the KaiA-B-C oscillator, with the compelling advantage of reduced model complexity. Theoretically, the small dynamical changes incurred due to omission of the above inter-conversions can be corrected by slight tuning of the phosphorylation/dephosphorylation rate constants of the original model.

The differential equations are numerically solved by MATLAB ODE solver. The parameters of basal phosphorylation/dephosphorylaiton rate constants are slightly modified based on the Rust *et al* model [1], and the rest of the parameters are chosen by hand to produce oscillations that satisfy the following criteria: (1) period is about 24 hr; (2) the amplitude of phosphorylated KaiC relative to total concentration of KaiC varies between ~20-60% [1]; (3) the relative amplitudes and phases of the four phospho-forms obey what observed in [1]. The parameters thus obtained are listed in supporting Table S1.

**Perturbation of the model of KaiABC oscillator with different concentrations of Kai proteins**

Nakajima *et al* experimentally observe the performance of the in vitro KaiABC oscillator under various combinations of Kai protein concentrations [9]. First, they perturb the total concentration of either KaiA or KaiB, while keeping the other two Kai proteins at the standard condition (i.e. [KaiA]^T^ = 1.2µM, [KaiB]^T^ = 3.5µM, [KaiC]^T^ = 3.5µM). They find that the oscillation of KaiC phosphorylation is stably observed in the presence of 0.6-2.46 µM KaiA while it becomes damped in the range of 3.6-6.0 µM KaiA. The concentration of KaiB, on the other hand, shows less impact on the oscillation in that the rhythm of KaiC phosphorylation persisted as long as the concentration of KaiB is above 1.75 µM. In addition, the amplitude and period of KaiC oscillation exhibit higher degree of variation under the perturbation of KaiA concentration, comparing to the almost unchanged amplitude when the concentration of KaiB is above 3.5 µM. Our computer simulation of the amplitude variation of the KaiABC oscillator in the 2-dimensional [KaiA]^T^-[KaiB]^T^ parameter plane agrees well with the experimental observations (Figure S2 A). Specifically, stable oscillation of the model exists in a long strip-shaped region parallel to the [KaiB]^T^-axis and the heat maps show that the amplitude and period of KaiC phosphorylation rhythm are tunable along the parameter-axis of KaiA concentration while quite invariant along the axis of KaiB concentration (Figure S2 A and D). Quantitatively, the model stably oscillates between the boundary of ~0.5-2.0 µM KaiA across the plane and above ~1.4 µM KaiB at the standard condition, showing a smaller oscillatory region than the experimental measurements. This is likely because that at some boundary conditions in the experiments damped rhythms were counted into the region of oscillation (e.g. at 3.6 µM KaiA, and at 0.875 µM KaiB).

Nakajima *et al* also perturb the concentrations of KaiB and KaiC simultaneously while keeping their ratio to 1:1 and find that the rhythms are observed within ~fourfold alteration of KaiB and KaiC concentration level [9]. Our simulations of amplitude and period variations in the [KaiB]^T^-[KaiC]^T^ parameter plane show that at the standard concentration of KaiA the stable oscillation persists in the region of ~2.9-6.6 µM of KaiB and KaiC, which is ~2.3 fold of change (Figure S2 B and E). Again, this smaller predicted oscillatory region is probably due to discrepancy in the definition of oscillation under boundary conditions (e.g. the decayed oscillation of KaiC phosphorylation at 7.0 µM and 10.5 µM of KaiB and KaiC are defined to be oscillatory by experiments but would not be counted as stable oscillation by computation). In addition, the simulated oscillatory region in [KaiB]^T^-[KaiC]^T^ plane predicts that the amplitude of oscillation is indeed robust with respect to the change of KaiB concentration, as depicted by the long strip-shaped oscillatory region parallel to the axis of KaiB, but is tunable by the concentration of KaiC. Note that the model proposed in [10] shows similar robustness to KaiB concentration when both KaiA and KaiB concentrations are perturbed.

Furthermore, our computation predicts that in the [KaiA]^T^-[KaiC]^T^ parameter plane the simulated oscillatory region is almost symmetrically located along some anti-diagonal direction, indicating that the variations of amplitude and period with respect to the concentration changes of KaiA and KaiC is linearly correlated (Figure S2 C and F). Such system behavior has not been predicted by other models.

**Robustness analysis** **of the model of KaiABC oscillator**

Bifurcation analysis is a tool in dynamical systems theory to study qualitative behavior of nonlinear systems [11]. For an autonomous oscillatory system that presents stable periodic solution, bifurcation analysis can identify parameter perturbations that destabilize oscillation to steady state and therefore has been used to quantify the robustness of oscillatory models [12,13]. Here we study the robustness of our model using bifurcation analysis with respect to the perturbation of the basal phosphorylation/dephosphorylation rate constants. Bifurcation diagrams show that oscillation persists in wide ranges of parameter values (Figure S3). Detailed information of the oscillation range for each of the perturbed parameters indicates that the rhythmic activity can endure at least ~10 fold of variation for the perturbed parameters (Table S2). Such robust analysis has not been reported for other models.

**Stochastic simulations of the** **model of KaiABC oscillator**

To study the behavior of the circadian clock under intrinsic noise, the model is simulated by the Gillespie algorithm [14], assuming that there are 2000-6000 number of Kai protein molecules in a cyanobacterial cell [15]. The stochastic simulations show that the circadian rhythm of the different phospho-forms of KaiC persists quite robustly with respect to the intrinsic noise (Figure S4). For the 10 runs of stochastic simulations of the total phosphorylated KaiC shown in Figure S8, the amplitude is 0.424±0.023 (percent) and the period is 23.9±1.4 (hr).

**References:**

1. Rust MJ, Markson JS, Lane WS, Fisher DS, O'Shea EK (2007) Ordered phosphorylation governs oscillation of a three-protein circadian clock. Science 318: 809-812.

2. Nishiwaki T, Satomi Y, Kitayama Y, Terauchi K, Kiyohara R, et al. (2007) A sequential program of dual phosphorylation of KaiC as a basis for circadian rhythm in cyanobacteria. Embo J 26: 4029-4037.

3. Hitomi K, Oyama T, Han S, Arvai AS, Getzoff ED (2005) Tetrameric architecture of the circadian clock protein KaiB. A novel interface for intermolecular interactions and its impact on the circadian rhythm. J Biol Chem 280: 19127-19135.

4. Iwase R, Imada K, Hayashi F, Uzumaki T, Morishita M, et al. (2005) Functionally important substructures of circadian clock protein KaiB in a unique tetramer complex. J Biol Chem 280: 43141-43149.

5. Akiyama S, Nohara A, Ito K, Maeda Y (2008) Assembly and disassembly dynamics of the cyanobacterial periodosome. Mol Cell 29: 703-716.

6. Brettschneider C, Rose RJ, Hertel S, Axmann IM, Heck AJ, et al. (2010) A sequestration feedback determines dynamics and temperature entrainment of the KaiABC circadian clock. Mol Syst Biol 6: 389.

7. Pattanayek R, Williams DR, Pattanayek S, Mori T, Johnson CH, et al. (2008) Structural model of the circadian clock KaiB-KaiC complex and mechanism for modulation of KaiC phosphorylation. Embo J 27: 1767-1778.

8. Kageyama H, Nishiwaki T, Nakajima M, Iwasaki H, Oyama T, et al. (2006) Cyanobacterial circadian pacemaker: Kai protein complex dynamics in the KaiC phosphorylation cycle in vitro. Mol Cell 23: 161-171.

9. Nakajima M, Ito H, Kondo T (2010) In vitro regulation of circadian phosphorylation rhythm of cyanobacterial clock protein KaiC by KaiA and KaiB. FEBS Lett 584: 898-902.

10. van Zon JS, Lubensky DK, Altena PR, ten Wolde PR (2007) An allosteric model of circadian KaiC phosphorylation. Proc Natl Acad Sci U S A 104: 7420-7425.

11. Strogatz S (2001) Nonlinear dynamics and chaos: with applications to physics, biology, chemistry and engineering: Westview Press.

12. Borisuk MT, Tyson JJ (1998) Bifurcation analysis of a model of mitotic control in frog eggs. J Theor Biol 195: 69-85.

13. Ma L, Iglesias PA (2002) Quantifying robustness of biochemical network models. BMC Bioinformatics 3: 38.

14. Gillespie DT (1977) Exact Stochastic Simulation of Coupled Chemical Reactions. J Phys Chem 81: 2340-2361.

15. Hafner M, Koeppl H, Hasler M, Wagner A (2009) 'Glocal' robustness analysis and model discrimination for circadian oscillators. PLoS Comput Biol 5: e1000534.
