# Supplementary figures and images for: Quantifying the Rhythm of KaiB-C Interaction for In Vitro Cyanobacterial Circadian Clock
Source: PLoS One. 2012 Aug 10;7(8):e42581. doi: 10.1371/journal.pone.0042581 (PMC3416856; doi:10.1371/journal.pone.0042581)

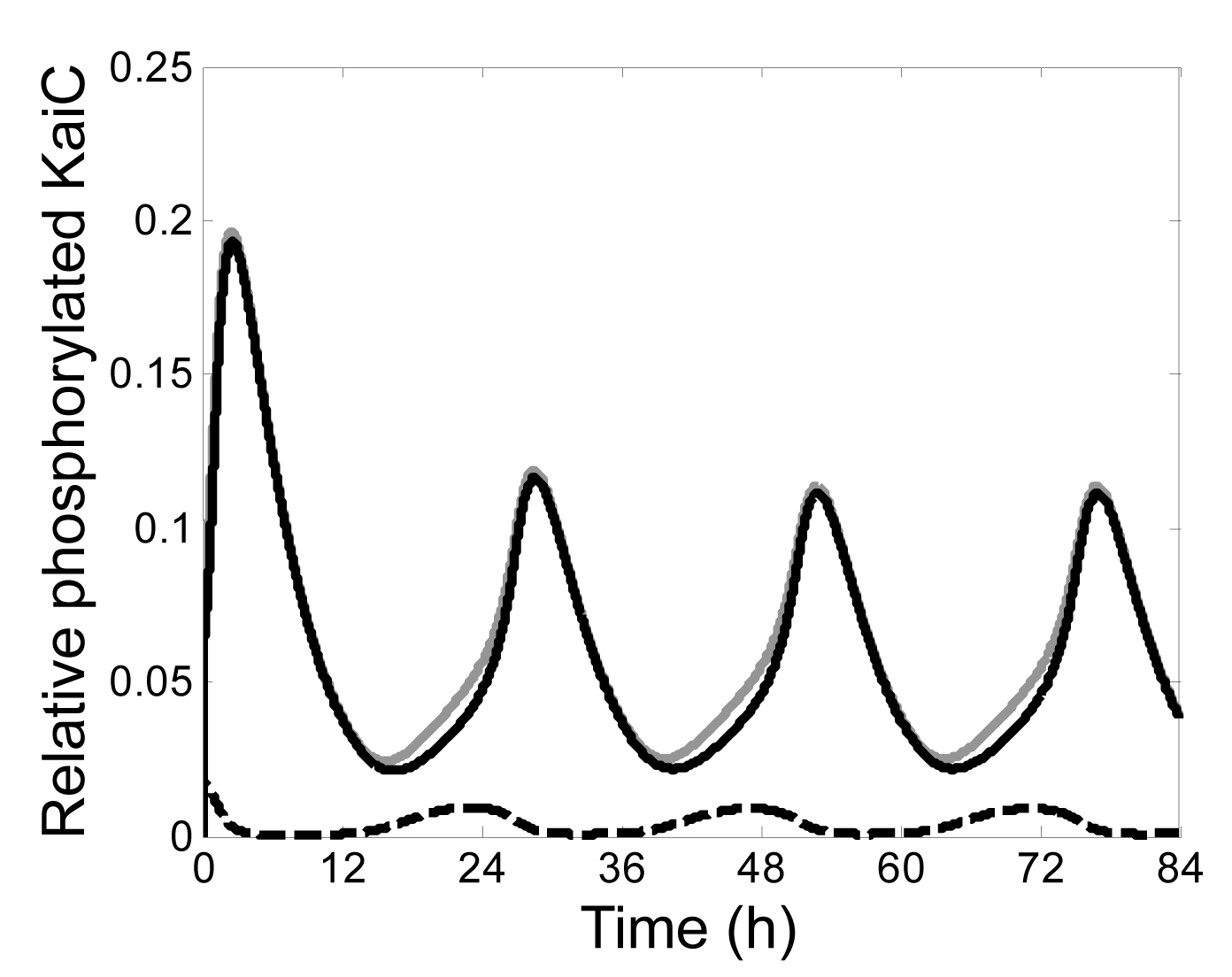

Supplement: Figure S1 — Oscillation of KaiB-S and KaiB-ST. Simulation of the complex KaiB-C (grey), the complex KaiB-S (solid black) and the complex KaiB-ST (dashed black). It shows that the amplitude of the complex KaiB-S is much higher than the amplitude of KaiB-ST. That is, the quantity of KaiB-C is mainly contributed by KaiBC-S rather than KaiBC-ST, which agrees with previous experimental findings that KaiB binds to KaiC only when the S-state of KaiC is abundant. (TIF) [file pone.0042581.s002.tif]

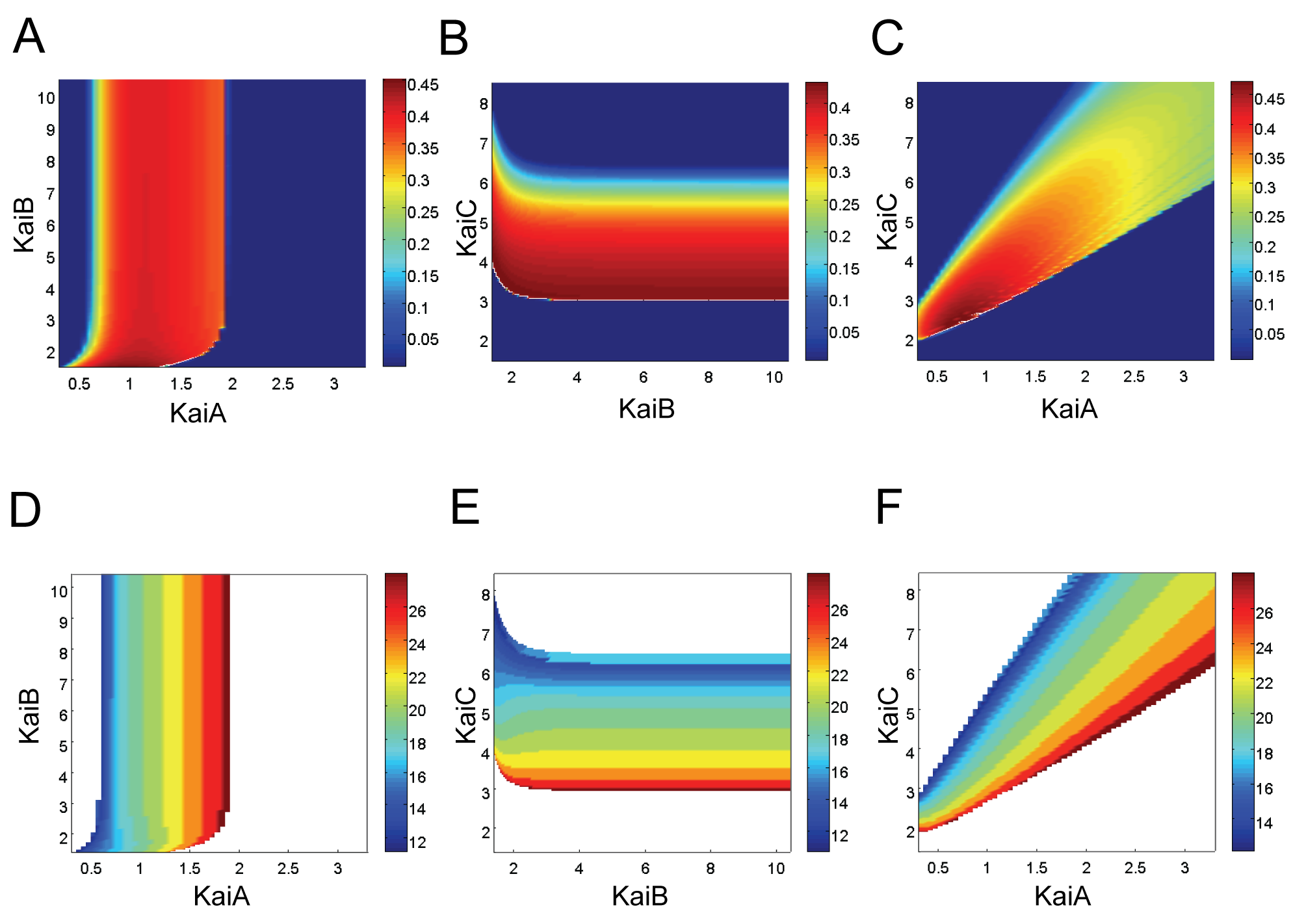

Supplement: Figure S2 — The amplitude and period of KaiC oscillation in the parameter planes of Kai protein concentrations. Simulations of the amplitude and period of the oscillation of KaiC phosphorylation are plotted in different 2-dimensional parameter planes when the total concentrations (unit: µM) of two Kai proteins are varied while the third protein concentration is maintained at the standard condition. Amplitude in (A) [KaiA]T-[KaiB]T (B) [KaiB]T -[KaiC]T and (C) [KaiA]T-[KaiC]T planes. Period in (D) [KaiA]T-[KaiB]T (E) [KaiB]T -[KaiC]T and (F) [KaiA]T-[KaiC]T planes. In each plot, the heat map represents the amplitude of phosphorylated KaiC relative to the total KaiC concentration, or period (unit: hour) of phosphorylated KaiC, with the corresponding color bar located to the right. (TIF) [file pone.0042581.s003.tif]

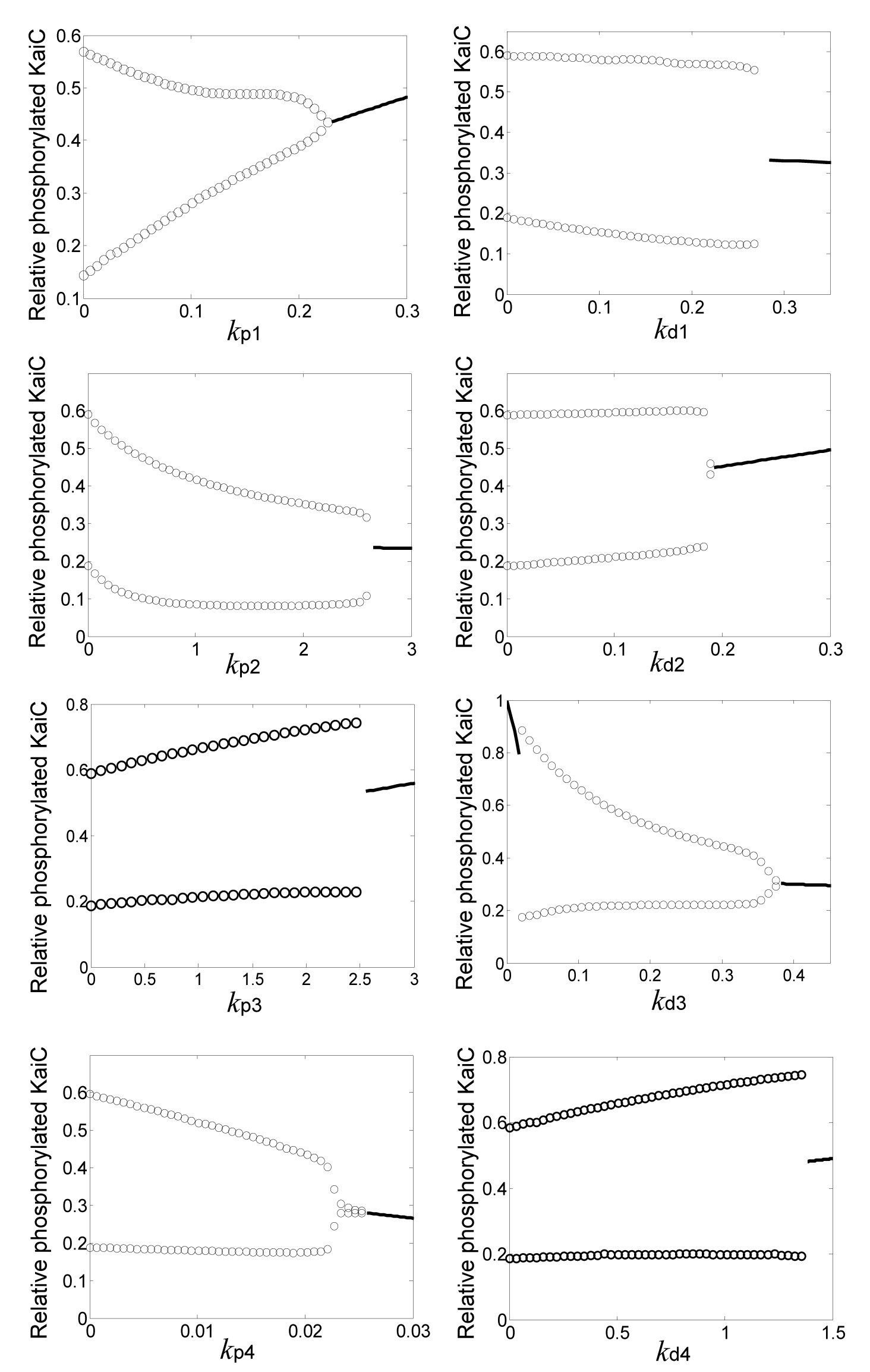

Supplement: Figure S3 — Bifurcation diagrams of the model of KaiABC oscillator. In each diagram, steady-state behavior of the system is plotted vs. the parameter under perturbation. Stable oscillation is represented by a pair of circles, indicating the maximum and minimum values of oscillation. Stable equilibrium point is represented by solid line. (TIF) [file pone.0042581.s004.tif]

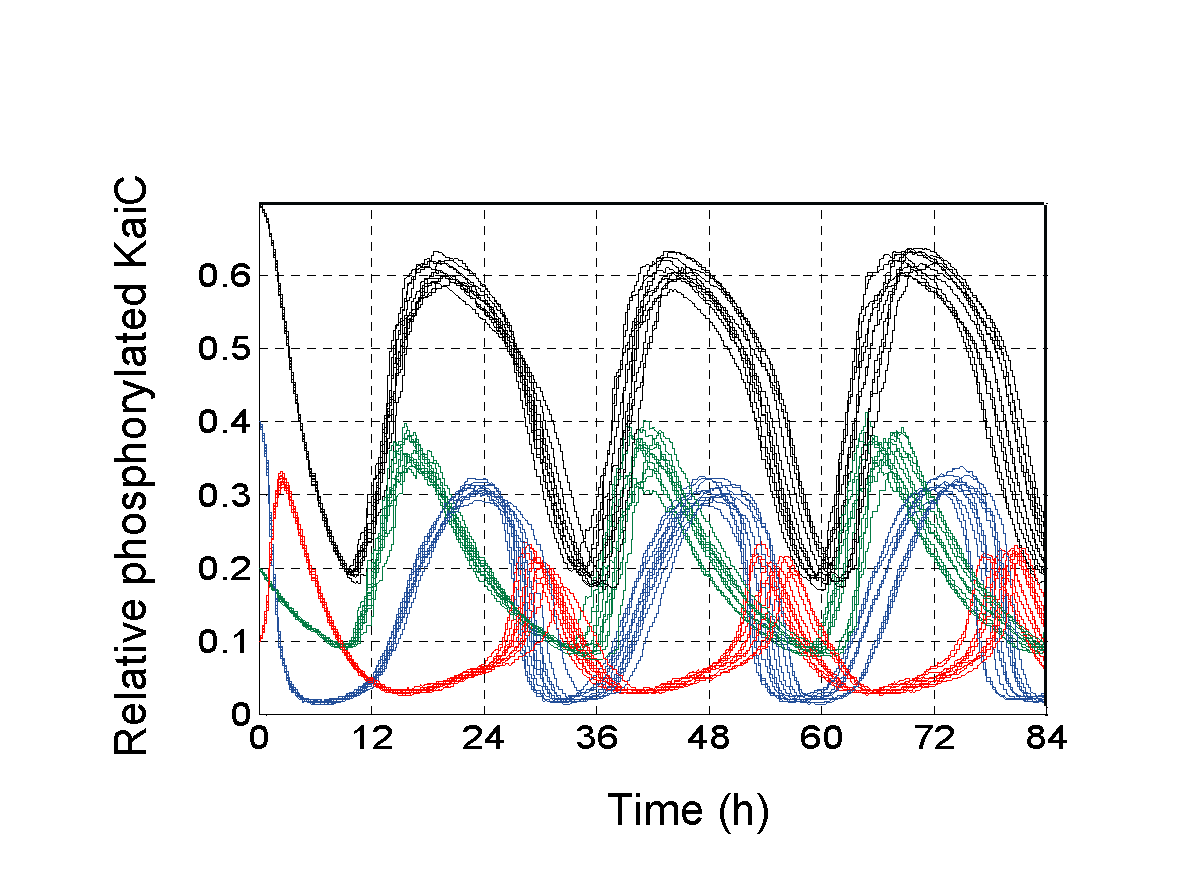

Supplement: Figure S4 — Stochastic simulations of KaiABC oscillator. The model is simulated by Gillespie algorithm. Shown above are 10 runs of stochastic simulation, where the total phosphorylated KaiC (black), T (green), ST(blue) and S (red) exhibit robust oscillations under noise. (TIF) [file pone.0042581.s005.tif]

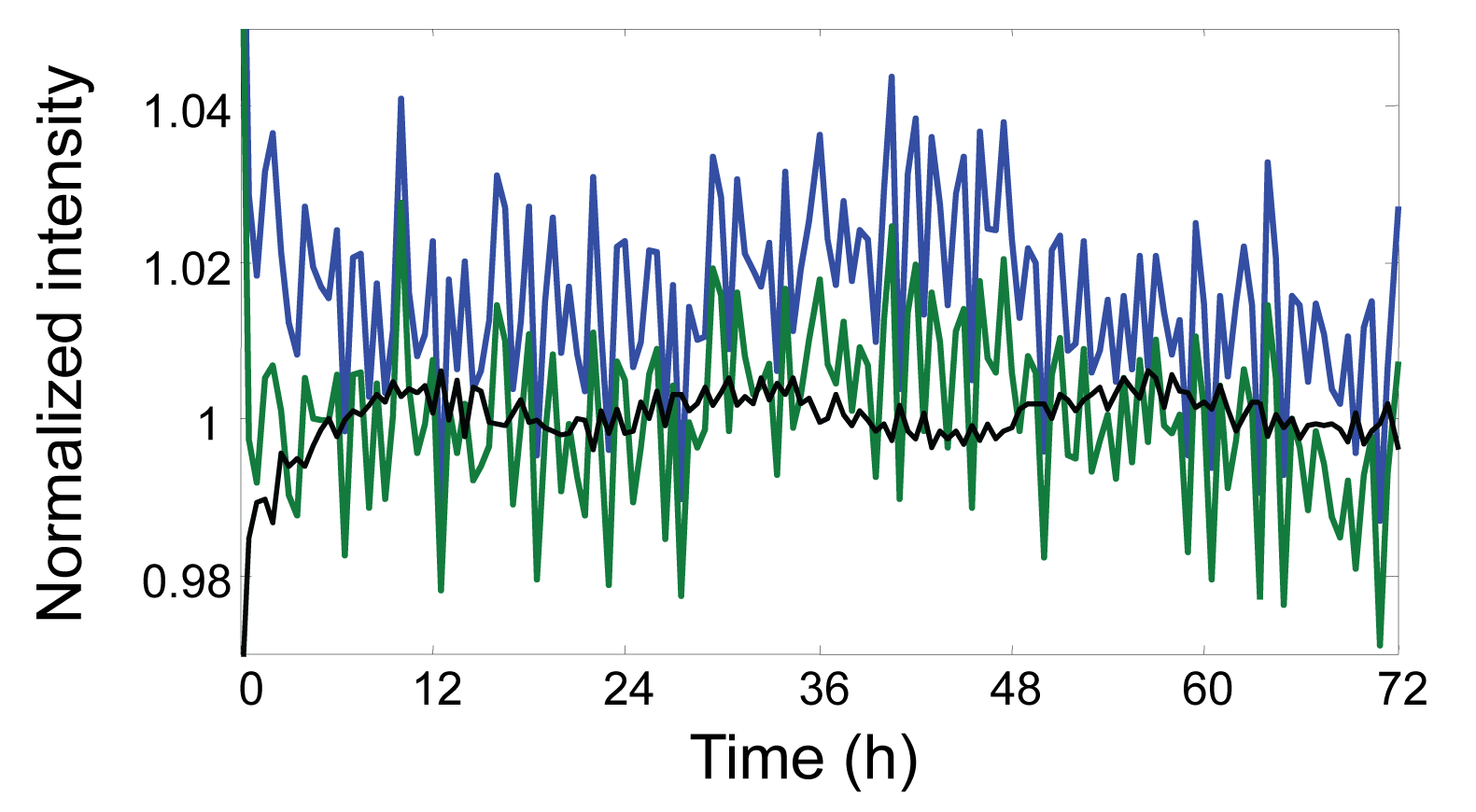

Supplement: Figure S5 — Time trajectories of donor and acceptor fluorescence intensities during FRET experiment. Exemplary time trajectories of CFP (blue curve) and YFP (green curve) fluorescence intensities normalized by their respective all-time-point mean values from a FRET experiment. The FRET signal obtained by YFP and CFP ratio normalized by its all-time-point mean value is shown as black solid line. There is significant common noise in the CFP and YFP data, while their ratio eliminates the noise and reveal the signal to a great degree. (TIF) [file pone.0042581.s006.tif]

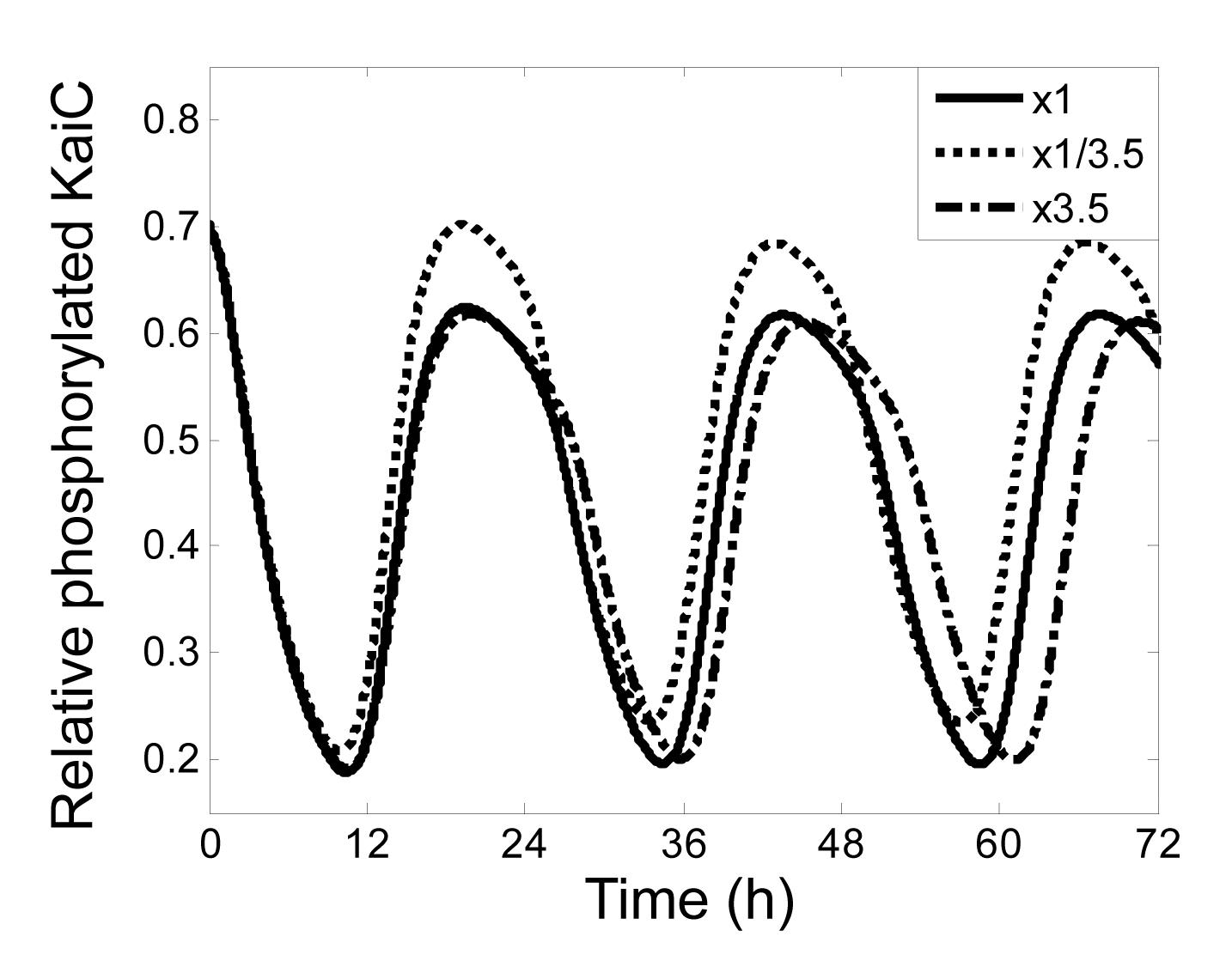

Supplement: Figure S6 — Temperature-compensation of model oscillations. When the dissociation constants for all binding reactions are simultaneously varied by a factor of ∼10, the period of KaiC phosphorylation changes by ∼10%. (TIF) [file pone.0042581.s007.tif]

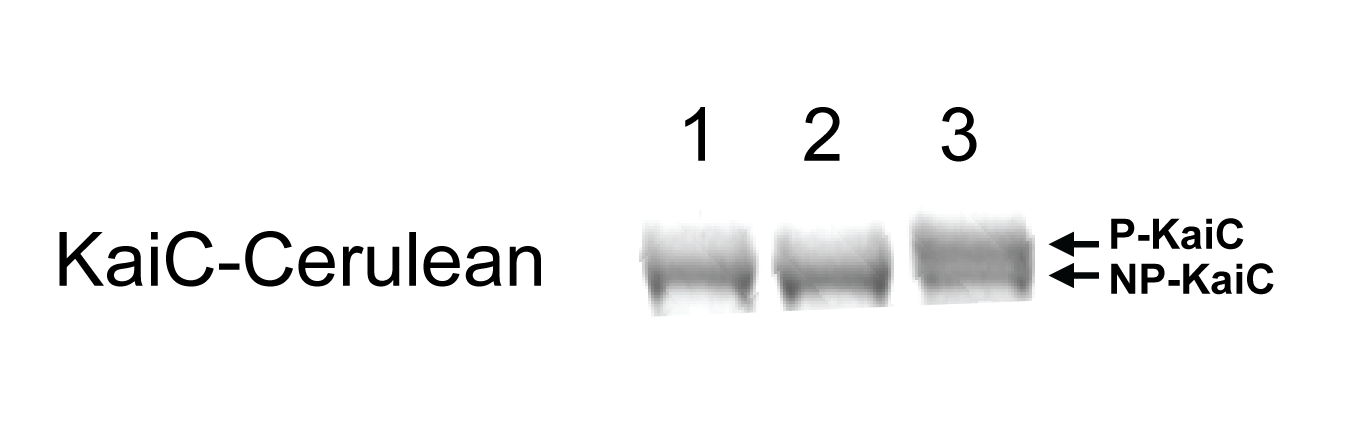

Supplement: Figure S7 — Phosphorylation test of KaiC-Cerulean with other Kai proteins. Gel electrophoresis of KaiC-Cerulean incubated alone (lane 1), with KaiB-Venus (lane 2) or with wild type KaiA (lane 3) overnight at 30°C in standard clock reaction buffer. The upper band represents phosphorylated KaiC (P-KaiC) and the lower band represents non-phosporylated KaiC (NP-KaiC). (TIF) [file pone.0042581.s008.tif]

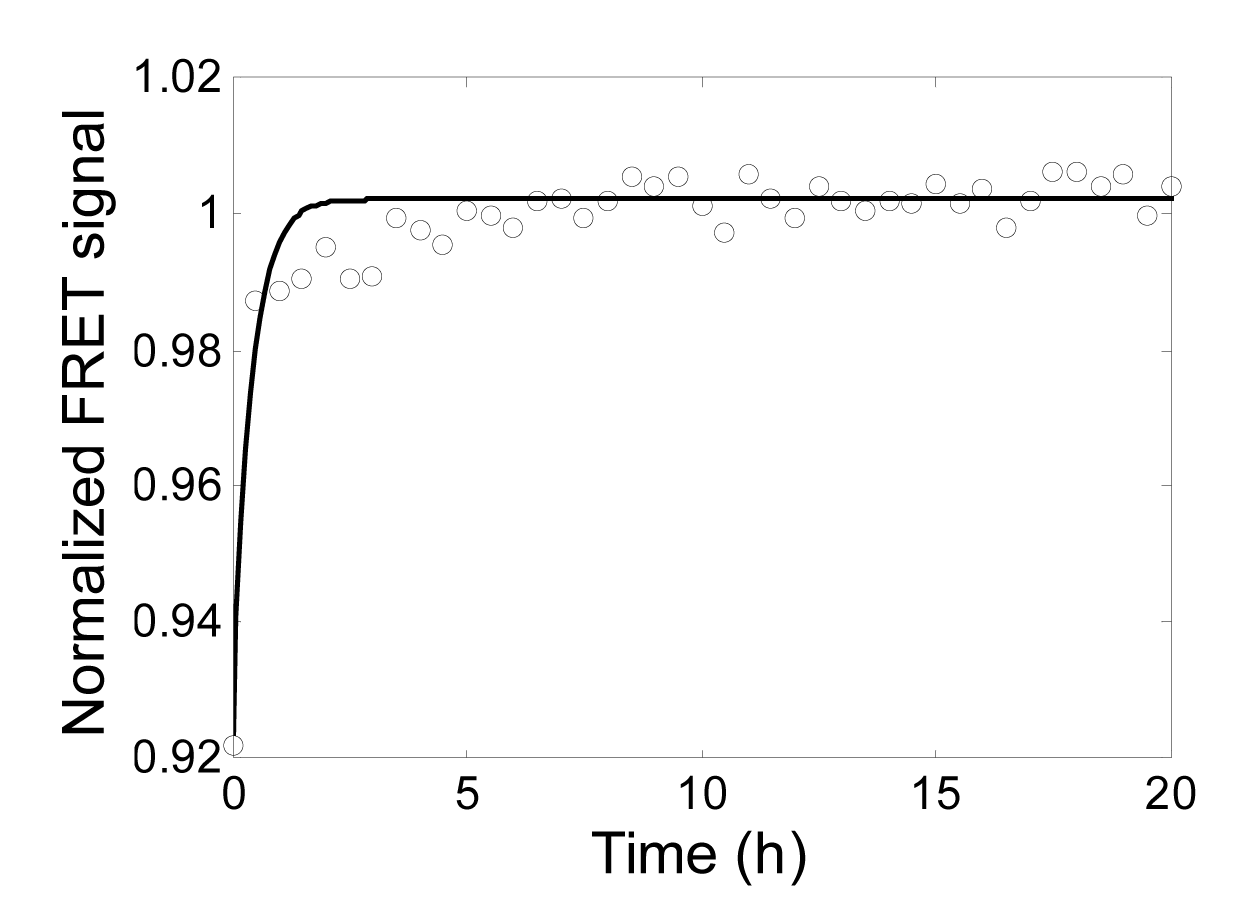

Supplement: Figure S8 — FRET trajectory of mixture of KaiC-Cerulean and KaiB-Venus. Time course of FRET signal when KaiC-Cerulean and KaiB-Venus were incubated in the standard clock reaction buffer at 30°C. The black circles represent an average curve of five normalized FRET trajectories. The black line represents fitted curve of the data. This plot shows that there is an abrupt rise within the first 1.5 hr. After this transient phase, the binding kinetics settles to a stable state. (TIF) [file pone.0042581.s009.tif]
