# Supplementary material for: Quantifying the Rhythm of KaiB-C Interaction for In Vitro Cyanobacterial Circadian Clock
Source: PLoS One. 2012 Aug 10;7(8):e42581. doi: 10.1371/journal.pone.0042581 (PMC3416856; doi:10.1371/journal.pone.0042581)
Supplement: Table S1 — Parameters for the model of KaiABC oscillator. (DOCX) [file pone.0042581.s010.docx]

| **Parameter** | **Description** | **Units** | **Constant** |
| --- | --- | --- | --- |
| *k_p1_* | Basal phosphorylation rate of U to T | hr^-1^ | 1.7×10^−4^ |
| *k_d1_* | Basal dephosphorylation rate of T to U | hr ^-1^ | 1.7×10^−3^ |
| *k_p2_* | Basal phosphorylation rate of T to ST | hr ^-1^ | 5×10^−4^ |
| *k_d2_* | Basal dephosphorylation rate of ST to T | hr ^-1^ | 1.7×10^−3^ |
| *k_p3_* | Basal phosphorylation rate of S to ST | hr^-1^ | 1.7×10^−3^ |
| *k_d3_* | Basal dephosphorylation rate of ST to S | hr ^-1^ | 0.067 |
| *k_p4_* | Basal phosphorylation rate of U to S | hr ^-1^ | 5×10^−4^ |
| *k_d4_* | Basal dephosphorylation rate of S to U | hr ^-1^ | 0.017 |
| *k_aa1_* | Association rate between U and KaiA | μM^-1^hr^-1^ | 57.6 |
| *k_ad1_* | Dissociation rate of UA | μM^-1^hr^-1^ | 19.2 |
| *k_aa2_* | Association rate between T and KaiA | μM^-1^hr^-1^ | 28.8 |
| *k_ad2_* | Dissociation rate of TA | μM^-1^hr^-1^ | 19.2 |
| *k_aa3_* | Association rate between ST and KaiA | μM^-1^hr^-1^ | 19.2 |
| *k_ad3_* | Dissociation rate of STA | μM^-1^hr^-1^ | 57.6 |
| *k_aa4_* | Association rate between S and KaiA | μM^-1^hr^-1^ | 19.2 |
| *k_ad4_* | Dissociation rate of SA | μM^-1^hr^-1^ | 24 |
| *k_ba1_* | Association rate between STA and KaiB | μM^-1^hr^-1^ | 28.8 |
| *k_bd1_* | Dissociation rate of STAB | μM^-1^hr^-1^ | 38.4 |
| *k_ba2_* | Association rate between SA and KaiB | μM^-1^hr^-1^ | 24 |
| *k_bd2_* | Dissociation rate of SAB | μM^-1^hr^-1^ | 24 |
| *k_ba3_* | Association rate between S and KaiB | μM^-1^hr^-1^ | 38.4 |
| *k_bd3_* | Dissociation rate of SB | μM^-1^hr^-1^ | 19.2 |
| *k_11_* | Phosphorylation rate of U facilitated by KaiA | hr ^-1^ | 1.44 |
| *k_21_* | Phosphorylation rate of T facilitated by KaiA | hr ^-1^ | 0.22 |
| *k_41_* | Phosphorylation rate of S facilitated by KaiA | hr ^-1^ | 0.24 |
| *k_42_* | Dephosphorylation rate of SAB to S | hr ^-1^ | 1.44 |
| *k_43_* | Dephosphorylation rate of ST facilitated by SB | hr ^-1^ | 1.44 |
| *k_44_* | Dephosphorylation rate of S facilitated by SB | hr ^-1^ | 0.86 |
| *K_MA1_* | Threshold conc. for UA-dependent phosphorylation of U | μM | 0.1 |
| *K_MA2_* | Threshold conc. for TA-dependent phosphorylation of T | μM | 0.3 |
| *K_MA3_* | Threshold conc. for SA-dependent phosphorylation of TS | μM | 0.6 |
| *K_MB1_* | Threshold conc. for SAB-dependent phosphorylation of U | μM | 0.004 |
| *K_MB2_* | Threshold conc. for SB-dependent dephosphorylation of ST | μM | 0.317 |
| *K_MB3_* | Threshold conc. for SB-dependent dephosphorylation of S | μM | 0.032 |
| *K_B1_* | Threshold conc. for cooperative binding of KaiB with STA | μM | 1.19 |
| *K_B2_* | Threshold conc. for cooperative binding of KaiB with SA | μM | 0.94 |
| *K_B3_* | Threshold conc. for cooperative binding of KaiB with S | μM | 1.06 |
