# Supplementary material for: Quantifying the Rhythm of KaiB-C Interaction for In Vitro Cyanobacterial Circadian Clock
Source: PLoS One. 2012 Aug 10;7(8):e42581. doi: 10.1371/journal.pone.0042581 (PMC3416856; doi:10.1371/journal.pone.0042581)
Supplement: Table S2 — Oscillation range for the model of KaiABC oscillator. The following model parameters are perturbed one at a time. The lower bound and upper bound of each of the parameters, within which oscillation exists, are listed in the table. The fold range is defined as the upper bound value divided by the lower bound value. (DOCX) [file pone.0042581.s011.docx]

| **Parameter** | **Units** | **Nominal Value** | **Lower bound value** | **Upper bound value** | **Fold range** |
| --- | --- | --- | --- | --- | --- |
| *k_p1_* | hr^-1^ | 1.7×10^−4^ | 0 | 0.23 | ∞ |
| *k_d1_* | hr ^-1^ | 1.7×10^−3^ | 0 | 0.27 | ∞ |
| *k_p2_* | hr ^-1^ | 5×10^−4^ | 0 | 2.6 | ∞ |
| *k_d2_* | hr ^-1^ | 1.7×10^−3^ | 0 | 0.19 | ∞ |
| *k_p3_* | hr^-1^ | 1.7×10^−3^ | 0 | 2.51 | ∞ |
| *k_d3_* | hr ^-1^ | 0.067 | 0.019 | 0.38 | 20 |
| *k_p4_* | hr ^-1^ | 5×10^−4^ | 0 | 0.024 | ∞ |
| *k_d4_* | hr ^-1^ | 0.017 | 0 | 1.36 | ∞ |
